# SORL1 variant links endosomal amyloid precursor protein mis‐sorting to axonal transport defects and neuronal dysfunction

**DOI:** 10.1002/alz.71782

**Published:** 2026-08-22

**Authors:** Miriam Lep, Klara Plesingrova, Jiri Sedmik, Sona Cesnarikova, Monica Feole, Ondrej Bernatik, Victorio Martin Pozo Devoto, Hana Hribkova, Katerina Amruz Cerna, Petr Fojtik, Nadezda Vaskovicova, Simona Bartova, Veronika Pospisilova, Tereza Vanova, Olav Michael Andersen, Dasa Bohaciakova, Jan Raska

**Affiliations:** ^1^ Faculty of Medicine, Department of Histology and Embryology Masaryk University Brno Czech Republic; ^2^ International Clinical Research Center (ICRC) St. Anne's University Hospital Brno Czech Republic; ^3^ Department of Biomedicine Aarhus University Aarhus Denmark

**Keywords:** Alzheimer's disease, axonal transport, endosomal trafficking, iPSCs, neuronal electrophysiology, *SORL1*

## Abstract

**INTRODUCTION:**

*SORL1* encodes the sorting receptor SORLA, a major genetic contributor to Alzheimer's disease (AD). Although *SORL1* loss disrupts endosomal trafficking and promotes amyloidogenic amyloid precursor protein (APP) processing, the functional consequences of specific missense variants in human neurons remain unclear.

**METHODS:**

We used isogenic induced pluripotent stem cell (iPSC)‐derived NGN2 neurons and 3D cerebral organoids carrying wild‐type, *SORL1* p.Y1816C knock‐in, or *SORL1* knockout alleles. We analyzed SORLA maturation and shedding, APP localization, amyloid‐β (Aβ) secretion, endosomal morphology, axonal transport of Rab5+ endosomes and APP, and network activity.

**RESULTS:**

The p.Y1816C variant impaired SORLA maturation and shedding and, together with knockout, caused enlarged early endosomes, APP retention, elevated Aβ secretion, amyloid deposition in organoids, axonal swellings, disrupted axonal transport, and neuronal hyperexcitability.

**CONCLUSIONS:**

The *SORL1* p.Y1816C variant is pathogenic in human neurons, and we reveal novel roles for SORLA in axonal transport and neuronal excitability. These findings highlight endosomal trafficking disruption as a central mechanism in AD pathogenesis.

## Highlights

- p.Y1816C impairs SORLA maturation and ectodomain shedding.
- Loss of *SORL1* enlarges early endosomes and mis‐sorts amyloid precursor protein (APP).
- SORLA dysfunction disrupts axonal transport of Rab5+ endosomes and APP.
- Axonal transport defects cause axonal swellings and cargo stalling.
- *SORL1* loss increases neuronal firing, bursting, and network synchrony.

## 1 BACKGROUND

Alzheimer's disease (AD), the leading cause of dementia, is characterized by amyloid‐β (Aβ) plaques and Tau tangles, but growing evidence points to early intracellular trafficking defects as key drivers of pathogenesis. Endosomal dysfunction is now recognized as an early and robust AD feature, detectable before extracellular Aβ deposition.^1^, ^2^, ^3^, ^4^, ^5^, ^6^, ^7^, ^8^, ^9^ Among AD‐linked genes, *SORL1* has emerged as the fourth autosomal dominant gene alongside amyloid precursor protein (*APP)*, presenilin (*PSEN) 1*, and *PSEN2*.^10^, ^11^, ^12^ It encodes SORLA, a neuronal sorting receptor essential for the trafficking of multiple cargos, most notably APP. In healthy neurons, SORLA binds APP and directs it away from early endosomes, where β‐secretase (BACE1)‐mediated amyloidogenic processing occurs, thereby limiting the generation of toxic Aβ peptides.^10^, ^13^ Loss of SORLA function due to *SORL1* mutations leads to missorting of APP into BACE1‐containing endosomes, resulting in enhanced Aβ production and secretion.^10^, ^14^ These trafficking defects are often accompanied by a marked enlargement of early endosomes, a morphological feature found not only in *SORL1*‐deficient models but also in other familial AD mutations, supporting its relevance as an early and generalizable AD‐related phenotype.^7^, ^8^, ^14^, ^15^, ^16^, ^17^, ^18^, ^19^ As much of this knowledge derives from *SORL1* knock‐out (KO) studies or overexpression models, the functional consequences of individual missense *SORL1* variants found in patients remain poorly understood.

Notably, we have previously described that one such variant, p.Y1816C, segregates with AD in three unrelated families.^20^ It lies within the fibronectin type III (3Fn) domain of SORLA, which we found to be essential for dimerization and interaction with the retromer complex that mediates endosome‐to‐surface trafficking of APP.^21^, ^22^ We further provided evidence that the p.Y1816C mutant impairs SORLA homodimerization in the endosome, leading to decreased trafficking to the cell surface and less soluble SORLA (sSORLA) ectodomain shedding.^20^, ^21^ However, the broader impact of the p.Y1816C mutation on the maturation of SORLA at the endogenous level, trafficking dynamics, and functional role in APP processing within human neuronal and 3D models remained unanswered.

Importantly, neurons rely on the efficient long‐range transport of key cargoes, such as APP and Rab5‐positive (Rab5+) early endosomes, via microtubule‐based motor proteins.^2^, ^23^, ^24^ Disruptions in these processes are consistently observed in AD models, where axonal swellings containing vesicular accumulations appear in patient brains, animal models, and iPSC‐derived neurons with familial AD mutations.^2^, ^23^, ^25^, ^26^, ^27^ We recently showed that neurons carrying the APP Swedish mutation exhibit impaired vesicle motility and increased APP stalling, contributing to synaptic dysfunction.^14^ Despite these findings, the direct involvement of SORLA in axonal transport or maintenance of axonal integrity has not been defined.

In parallel, growing evidence links AD pathogenesis to early neuronal hyperexcitability. iPSC‐derived neurons with *APP* or *PSEN1* mutations display increased spontaneous activity, altered calcium dynamics, and network synchrony.^17^, ^18^, ^28^ Similar abnormalities, such as epileptiform discharges and increased seizure susceptibility, are also seen in 5xFAD and APP/PS1 mouse models.^29^, ^30^ Interestingly, Zhou *et al*.^31^ previously demonstrated that the APP Swedish mutation in iPSC‐derived neurons increases the number of functional synapses and strongly suggested that, at the physiological level, Aβ might be synaptogenic. Although SORLA is well established as a regulator of APP trafficking and Aβ production, its potential influence on neuronal excitability and network activity remains unclear. Aside from limited evidence of increased firing in *SORL1*‐deficient neurons,^18^, ^32^ the broader electrophysiological consequences of SORLA dysfunction have yet to be investigated.

To address these gaps, we generated isogenic human iPSC‐derived neurons and cerebral organoids carrying either the *SORL1* p.Y1816C variant or a *SORL1* knockout and examined their molecular, structural, and functional phenotypes. Our results show that the p.Y1816C variant impairs SORLA maturation and shedding and that SORLA dysfunction causes enlarged endosomes with APP retention, elevated Aβ secretion, disrupted axonal transport, neuritic swellings, and network hyperexcitability. Importantly, this is the first evidence that SORLA loss or mutation leads to axonal transport impairment and “trafficking clogging,” extending SORLA biology beyond soma‐restricted endosomal pathology. Organoids faithfully reproduced all major phenotypes, providing the first direct demonstration of AD‐like pathology driven by a patient‐derived *SORL1* mutation in 3D human brain tissue. Together, these findings establish SORLA as a key regulator of axonal trafficking and neuronal function and highlight endosomal transport defects as an early and actionable driver of AD pathology.

## 2 MATERIALS AND METHODS

For detailed materials and methods, see the Supplementary Materials and Methods. A comprehensive list of all used cell lines and clones is provided in Table S1; a list of all experimental samples, including iPSC clones used for individual analyses and the number of biological replicates (independent differentiations), is provided in Table S2; and media compositions, antibodies, sequences of guide RNAs (gRNAs), single‐stranded oligodeoxynucleotides (ssODNs), and polymerase chain reaction (PCR) primers are provided in Table S3.

**RESEARCH IN CONTEXT**

1. **Systematic review**: We reviewed prior literature using PubMed, Google Scholar, and conference abstracts focusing on SORL1 genetics, SORLA trafficking biology, endosomal dysfunction, axonal transport, and neuronal network phenotypes in Alzheimer's disease (AD). Existing work demonstrates that SORL1 mutations disrupt APP trafficking and increase amyloidogenic processing, but most studies relied on overexpression systems or limited functional readouts. Few studies have examined pathogenic SORL1 variants in human neurons or organoids, and virtually none addressed axonal transport or neuronal excitability.
2. **Interpretation**: Our data show that the SORL1 p.Y1816C variant and SORL1 loss disrupt SORLA maturation, enlarge early endosomes, impair axonal transport of Rab5+ endosomes and amyloid precursor protein (APP), increase amyloid‐β (Aβ) secretion, and induce neuronal hyperexcitability. These findings extend SORLA biology beyond somatic endosomes to axonal and network homeostasis.
3. **Future directions**: Key priorities include defining molecular mechanisms linking SORLA dysfunction to axonal transport, testing additional SORL1 variants across models, and determining whether correcting trafficking defects normalizes neuronal excitability and disease‐relevant phenotypes.

### 2.1 Cell culture and gene editing

A doxycycline‐inducible NGN2 iPSC line (i3N; RRID: CVCL_C7XJ) was used and maintained as described previously.^33^ Three *SORL1* knockout (KO) clones and one heterozygous p.Y1816C knock‐in (KI) clone were generated using CRISPR/Cas9 RNPs (Cas9–GFP [green fluorescent protein], Sigma). KO lines were produced by targeting exon 6 with a published gRNA^14^; the p.Y1816C KI was introduced by targeting exon 41 with a custom gRNA and ssODN, listed in Table S3. To control for editing‐related artifacts, two MOCK clones were generated by running the full CRISPR workflow without introducing a mutation. Unless indicated otherwise, experiments used three WT clones (parental + two MOCK), three KO clones, and one KI clone. In all graphs, clone 1 of each genotype is represented by circles, clone 2 by squares, and clone 3 by triangles.

### 2.2 Differentiation of 2D neurons and 3D cerebral organoids

2D Neuronal differentiation of i3N iPSCs was performed using a previously published protocol^33^ in Induction Medium (IM) supplemented with 2 µg/mL doxycycline (Sigma). On Day 3, cells were replated and samples were harvested on Days 20, 30, and 40. 3D cerebral organoids were generated using an unguided protocol from *Lancaster et al.* ^34^ as previously described by Vanova *et al.*,^35^ without doxycycline exposure and therefore without intentional induction of the integrated NGN2 transgene. Samples were collected on Days 9, 15, 30, and 60. For whole cell lysate and RNA isolation, 5 to 10 organoids were pooled per sample. For ELISA, individual organoids were cultured in 12‐well plates with 0.8‐1.5 mL of Essential 6 medium (Thermo Fisher Scientific) for 72 hours prior to the collection of conditioned media and organoids.

### 2.3 Immunocytochemistry and image analysis

iPSC‐derived neurons were cultured on glass coverslips, fixed with 3.7% paraformaldehyde (PFA), permeabilized, blocked, and incubated with primary antibodies overnight at 4°C followed by secondary antibodies for 1 hour at room temperature. Nuclei were counterstained with DAPI (4′,6‐diamidino‐2‐phenylindole) and coverslips were mounted with ProLong Gold (Thermo Fisher Scientific). Endosome enlargement analysis was performed using ImageJ. Neuronal regions were identified using MAP2‐derived mask, EEA1 puncta were segmented by thresholding, and quantified by automated particle analysis. A total of 28‐33 images per genotype were analyzed (three biological replicates).

For the analysis of Aβ in organoids, organoids were fixed in 3.7% PFA, embedded in agarose, vibratome‐sectioned (200 µm sections), and stained with primary antibodies for 4 days and secondary antibodies for 24 hours at 4°C, followed by DAPI staining and clearing. For the staining of neural markers and early endosomes in organoids, organoids were fixed in 3.7% PFA, cryoprotected in 30% sucrose solution, embedded in OCT and sectioned at 20 µm using a Leica CM1850 cryostat. Sections were stained with primary antibodies overnight at 4°C, followed by secondary antibodies for 1 hour at room temperature. Nuclei were counterstained with DAPI, and sections were mounted using ProLong Gold (Thermo Fisher Scientific). Confocal imaging was performed on Axio Observer.Z1 with an LSM 800 confocal unit and a 63× oil or 20× objective (Zeiss).

### 2.4 Proximity ligation assay

iPSC‐derived neurons were seeded on glass coverslips and processed for proximity ligation assay (PLA) using the Duolink^®^ In Situ Red Starter Kit (Sigma) according to the manufacturer's protocol. Primary antibodies (Table S3) were applied overnight at 4°C, followed by ligation and amplification steps. Cells were mounted with DAPI and imaged within 2 days. Confocal microscopy was performed on an Axio Observer.Z1 with an LSM800 and 63× oil objective (1024 × 1024 px). PLA puncta were quantified in ImageJ using Analyze Particles (size 10‐150 px; circularity 0‐1). A total of 39 images per genotype were analyzed (three biological replicates, 13 images each).

### 2.5 Live imaging and axonal transport analyses

iPSC‐derived neurons (60,000 cells/well, eight‐well ibidi slides) were transduced at Day 20 with APP–GFP lentivirus (MOI 5) and BacMam RFP‐Rab5a (MOI 10) in CNM medium. After 24 h, medium was replaced and cultures matured until live imaging at Day 30. Before acquisition, CNM was exchanged for phenol‐red–free artificial cerebrospinal fluid (aCSF). Time‐lapse imaging of axonal transport was performed at 37°C/5% CO_2_ on a Zeiss LSM 780 with a 63×/1.4 NA oil objective (60‐second recordings; APP–GFP at 2 fps, Rab5a at 1 fps).

Movies were pre‐processed in ImageJ and analyzed in Imaris v10 using “Spots” (APP–GFP) or “Surfaces” (Rab5a) tracking with autoregressive motion. Tracks shorter than 10 s were excluded. Particles with total displacement ≥ 250 nm were considered moving; movement direction defined anterograde vs. retrograde. Fast‐moving APP–GFP vesicles were classified by mean velocity (≥ 0.1 µm/s or ≤ –0.1 µm/s). Rab5a‐RFP vesicles were categorized as moving vs. stationary based on ≥ 250 nm displacement. Track displacement, duration, length, and particle size (Rab5a only) were extracted from Imaris output for downstream analysis.

### 2.6 Electrophysiology

iPSC‐derived neurons (20,000 cells/well) were plated on PLO/laminin‐coated 96‐well microelectrode array (MEA) plates (Axion BioSystems), 8 wells per clone, and maintained in CNM for 8 weeks. Spontaneous activity was recorded every other day for 5 minutes at 37°C using the Axion Maestro system. Raw signals were processed with Neural Metrics Tool (AxiS 2.4). Electrodes were considered active if > 1 spike/min; spikes were detected at 6.5 SD above noise; bursts were ≥5 spikes with ≤100 ms inter‐spike interval. Network activity was quantified using weighted mean firing rate, number of active electrodes, percentage of spikes in bursts, burst count, and synchrony index.

### 2.7 Data analysis

Data are presented as mean ± standard error of the mean (SEM). Normality of data distribution was assessed prior to statistical testing. Parametric tests (t‐test or one‐way analysis of variance [ANOVA]) were applied to normally distributed data, while non‐parametric tests (Mann–Whitney U test or Kruskal–Wallis test) were used for non‐normally distributed data when comparing two or three groups, respectively. Statistical analyses were performed using GraphPad Prism version 9.0.0 for Windows (GraphPad Software; www.graphpad.com). A *p*‐value ≤ 0.05 was considered statistically significant.

### 2.8 Data availability

This study does not contain any high‐throughput data dataset. All analyzed data are included in this manuscript. Raw data will be available upon reasonable request from the corresponding authors of this manuscript.

## 3 RESULTS

### 3.1 The pathogenic SORLA p.Y1816C variant impairs SORLA protein maturation in iPSCs

**FIGURE 1:**
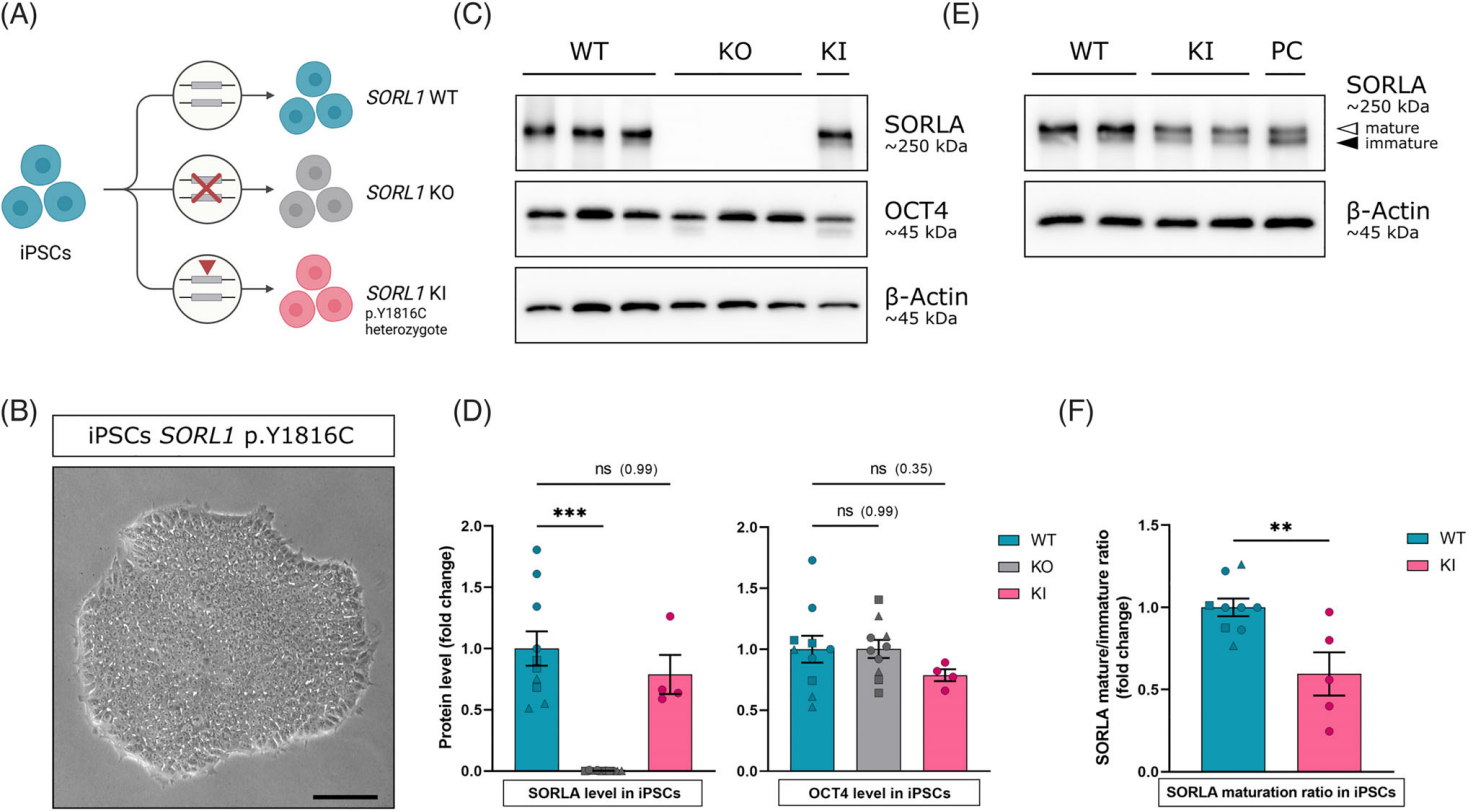
Characterization of isogenic iPSC lines carrying the *SORL1* p.Y1816C variant and analysis of SORLA protein maturation. (A) Schematic overview of the CRISPR/Cas9 gene editing strategy used to generate isogenic *SORL1* KI (heterozygous p.Y1816C) and KO iPSC lines. (B) Representative colony morphology of pluripotent iPSCs carrying the *SORL1* p.Y1816C variant. (C, D) Western blot analysis on 10% PAGE gel showing expression and densitometric quantification of OCT4 and total SORLA across WT, KI, and KO lines. All clones used in this study are shown. (E) Western blot on 6% PAGE gel visualizing the mature and immature forms of SORLA in WT and KI lines. PC indicates positive control cell line with compromised SORLA maturation.^16^ (F) Densitometric quantification of the ratio of mature to immature SORLA forms; β‐actin was used as a loading control. Each dot represents one biological replicate (n ≥ 4). Data are presented as mean ± SEM. Statistical significance was assessed using one‑way ANOVA followed by Dunnett's Tukey's multiple comparison test, Kruskal–Wallis followed by Dunn's multiple comparison test or t‐test; **p < 0.01, ***p < 0.001; Brightfield scale bar: 200 µm. A list of all experimental samples, including iPSC clones used for individual analyses and the number of biological replicates, is provided in Table S2. ANOVA, analysis of variance; iPSC, induced pluripotent stem cell; KI, knock‐in; KO, knock‐out; PAGE, polyacrylamide gel electrophoresis; SEM standard error of the mean; WT, wild‐type.

In this study, we used isogenic human iPSC lines carrying either wild‐type (WT) or heterozygous *SORL1* p.Y1816C variant, reflecting the genotype found in AD patients, hereafter referred to as knock‐in (KI). We also included *SORL1* KO line. Both KI and KO iPSCs were generated via CRISPR/Cas9 genome editing^20^ (Figure 1A). The pluripotent status of all lines was confirmed by characteristic colony morphology and stable expression of the pluripotency marker OCT4, which remained unchanged following gene editing (Figure 1B–D). All cell lines exhibited a normal karyotype and carried the apolipoprotein E (APOE) 3/3 genotype (Figure S1A,B). Genotyping PCR across the KO‐targeted region showed a reduced amplicon size in KO cells, consistent with a small deletion at the edited locus (Figure S1C). The presence of the correct point mutation in the *SORL1* gene was verified via Sanger sequencing (Figure S1D). No alterations were detected at the predicted off‐target loci, and no exon skipping of exon 41 was observed. (Figure S1E,F).

To evaluate the potential functional consequences of the p.Y1816C mutation, we next examined SORLA protein expression and maturation at the undifferentiated stem cell state of the cell lines. Given that SORLA undergoes essential post‐translational processing required for its intracellular trafficking and activity, we analyzed both the immature and mature forms of the protein. While the total SORLA protein levels from expression of both alleles remained unchanged in KI cells compared to WT controls (Figure 1C,D), we observed a pronounced reduction in the mature form of SORLA in the KI line (Figure 1E). Further densitometric analysis and quantification of the ratio between mature and immature SORLA forms revealed a significant decrease, resulting in approximately a 40% reduction in SORLA maturation (Figure 1F). Overall, these initial data demonstrate that the pathogenic *SORL1* p.Y1816C variant does not affect the pluripotency of generated iPSCs but leads to impaired processing of the SORLA protein in iPSCs. Given that reduced levels of mature, functional SORLA have been linked to disrupted endosomal trafficking and increased AD risk,^36^ our initial findings prompted us to further explore pathogenic mechanisms by which the *SORL1* p.Y1816C variant may contribute to disease development at a cellular level, particularly in neuronal models.

### 3.2 The SORL1 p.Y1816C variant reduces SORLA protein shedding in neurons and cerebral organoids

To further comprehensively assess the cellular consequences of the *SORL1* p.Y1816C mutation and SORLA deficiency in human neurons, we conducted parallel experiments using both 2D *NGN2*‐induced neurons and 3D cerebral organoids derived from isogenic iPSC lines. This combined approach allowed us to validate findings across simplified 2D neurons and neuronal networks, as well as more complex 3D neural systems that mimic human brain architecture and cellular interactions.^34^, ^35^

**FIGURE 2:**
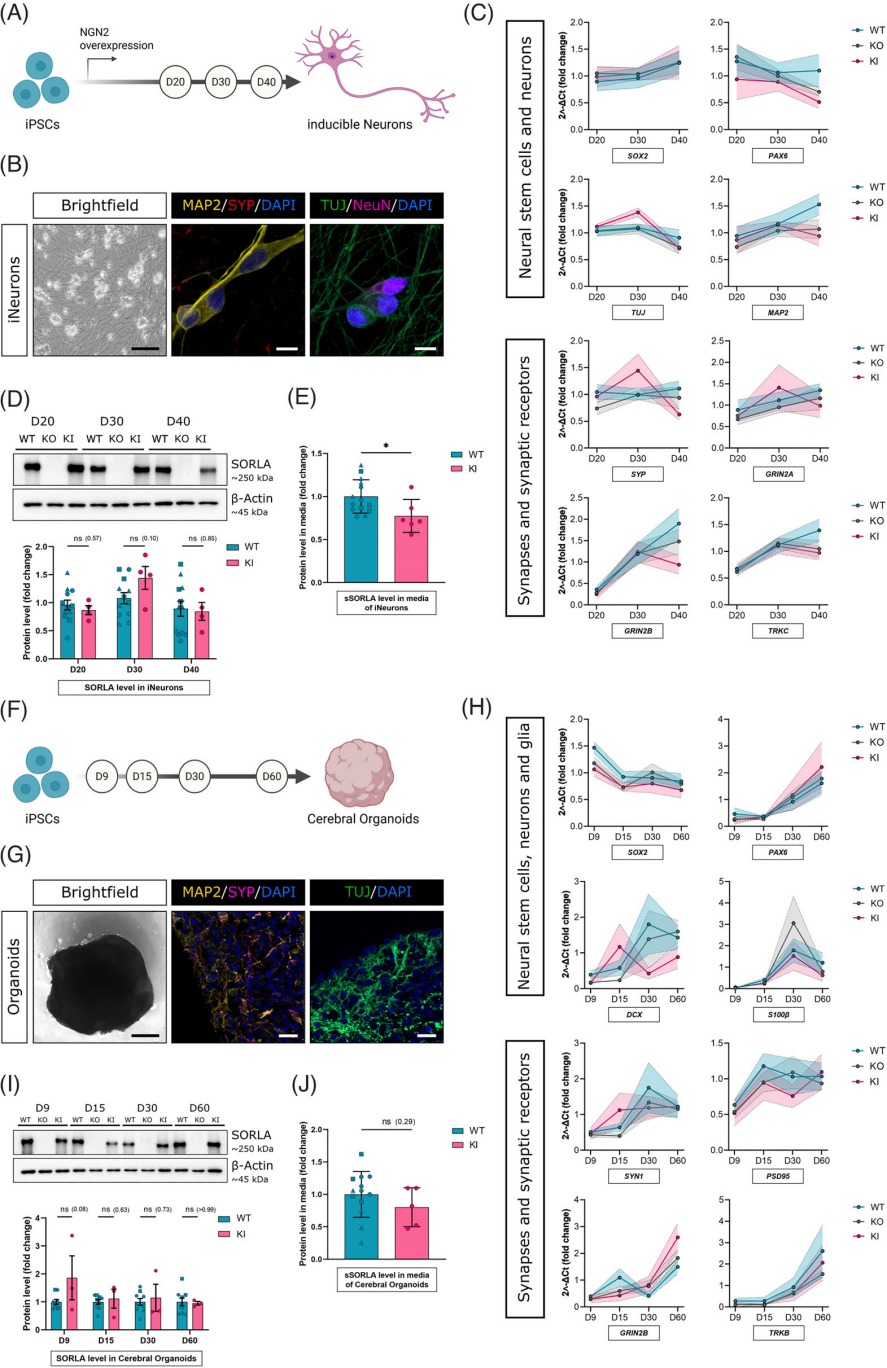
*SORL1* p.Y1816C variant affects SORLA shedding in 2D and 3D human models. (A) Schematic of the differentiation protocol for generating 2D neurons using a doxycycline‐inducible NGN2 overexpression system and sample collection; (B) Representative images showing neuronal differentiation of iPSC lines, with expression of TUJ, NeuN, MAP2, and SYP; (C) Gene expression analysis of neural stem cell (*SOX2*, *PAX6*), neuronal (*TUBB3*, *MAP2*), and synaptic markers and receptors [*SYP*, *GLUN2A*, *GLUN2B*, *NTRK3* (TRKC)]; (D) Western blot analysis showing levels of intracellular SORLA during neuronal differentiation in WT and KI lines; (E) ELISA analysis of sSORLA levels in the culture medium of WT‐ and KI‐derived neurons at Day 30; (F) Schematic of the protocol used for generating 3D cerebral organoids and timeline of sample collection; (G) Representative images showing Day 60 cerebral organoids with expression of mature neuronal markers (MAP2, SYP, NeuN); (H) Gene expression profiles of progenitor (*SOX2, PAX6*), neuronal (*DCX*), synaptic [(*SYN1, PSD96, GRIN2B, NTRK2* (TRKB)], and glial (*S100B*) markers across developmental progression of WT, KO, and KI lines. (I) Total SORLA protein expression compared between genotypes during organoid differentiation. (J) ELISA analysis of sSORLA in Day 60 organoid culture medium. Each dot in (C) and (H) represents the average 2˄‐ΔCt; each dot in (D), (E), (I), and (J) represents one biological replicate (n ≥ 3). Data are presented as mean ± SEM. Statistical significance was assessed using Mann–Whitney test or t‐test; *p < 0.05 was determined using an unpaired statistical test. Brightfield scale bar: 50 µm (B), 250 µm (G); Immunofluorescence scale bar: 10 µm (B), 20 µm (G). A list of all experimental samples, including iPSC clones used for individual analyses and the number of biological replicates, is provided in Table S2. ELISA, enzyme‐linked immunosorbent assay; iPSC, induced pluripotent stem cell; KI, knock‐in; KO, knock‐out; SEM, standard error of the mean; SYP, synaptophysin; WT, wild‐type.

For the generation of 2D neurons, we used a doxycycline‐inducible *NGN2* overexpression system, as previously described.^20^, ^33^ Neuronal differentiation was monitored at three time points, days 20, 30, and 40, to track developmental progression (Figure 2A). All iPSC lines successfully differentiated into neurons, forming characteristic 2D neuronal networks and expressing canonical neuronal markers including TUJ1, NeuN, MAP2, and synaptophysin (SYP) (Figure 2B). Subsequent extensive molecular characterization confirmed robust neuronal identity across all genotypes. While some variability in differentiation timing of gene or protein expression was observed between biological replicates, the expression of lineage‐ and function‐specific markers followed consistent patterns across experiments, irrespective of *SORL1* WT, KO, or KI genotypes. Specifically, we analyzed neural stem cell markers (SOX2, SOX1, PAX6), neuronal markers (DCX, TUJ1, MAP2, NeuN), synaptic markers (SYN1, SYP, PSD95), and synaptic receptor components (GLUA, GLUN1, GLUN2A, GLUN2B, TRKB, TRKC) at both RNA and/or protein levels, providing strong evidence of neuronal specification (Figure 2C and Figure S2A,B).

Importantly, we also assessed the expression and processing of SORLA during neuronal differentiation. Results show that intracellular SORLA protein levels remained comparable between WT and p.Y1816C mutant neurons (Figure 2D). However, quantification of shedded sSORLA in the culture medium at day 30 by ELISA revealed a significant reduction in mutant neurons compared to controls (Figure 2E). This decrease mirrors previously reported reductions in sSORLA levels in cerebrospinal fluid from human AD patients carrying *SORL1* mutations, further supporting the relevance of our in vitro model.^20^, ^37^

In parallel, 3D cerebral organoids were generated using a modified Lancaster protocol under non‐induced, patterning‐based differentiation conditions^34^, ^35^ and collected at four developmental stages (days 9, 15, 30, and 60; Figure 2F). By day 60, the organoids displayed typical morphology and regional organization and expressed mature neuronal markers, including MAP2, SYP, and NeuN (Figure 2G). Further analyses confirmed that the expression of pluripotency markers progressively declined during differentiation in 3D organoids, while progenitor, neuronal, synaptic, and glial markers followed expected developmental trajectories. Importantly, no consistent differences were observed between control, *SORL1* KO, or *SORL1* p.Y1816C variant lines when assessing both qPCR and Western blot results. (Figure 2H and Figure S2C,D). Notably, similarly to 2D neurons, total levels of SORLA protein detected in cerebral organoids over the course of differentiation remained comparable between analyzed cell lines (Figure 2I). However, sSORLA levels measured by ELISA in the day 60 culture medium showed a decreasing trend in mutant lines, consistent with 2D neuron data, though the difference did not reach statistical significance (Figure 2J). Two additional biological replicates analyzed by Western blot revealed a similar trend, with reduced sSORLA levels in KI‐derived organoids compared to WT controls (Figure S2E,F). Together, these findings demonstrate that the *SORL1* p.Y1816C variant impairs SORLA maturation in iPSC cultures and shedding in both 2D neuronal cultures and more physiologically relevant 3D cerebral organoids, highlighting a consistent defect in SORLA processing across complementary and reinforcing human‐based models expressing the pathogenic mutation endogenously.

### 3.3 p.Y1816C induces enlargement of early endosomes in both 2D neurons and 3D organoids

Thus far, our data have demonstrated that the *SORL1* p.Y1816C variant alters the biology of the SORLA protein itself, leading to impaired maturation and reduced shedding. To further assess the impact of this variant on neuronal physiology, we next investigated the effect of reduced SORLA activity on endosomal biology, a process previously associated with SORLA dysfunction.^9^, ^14^, ^20^

**FIGURE 3:**
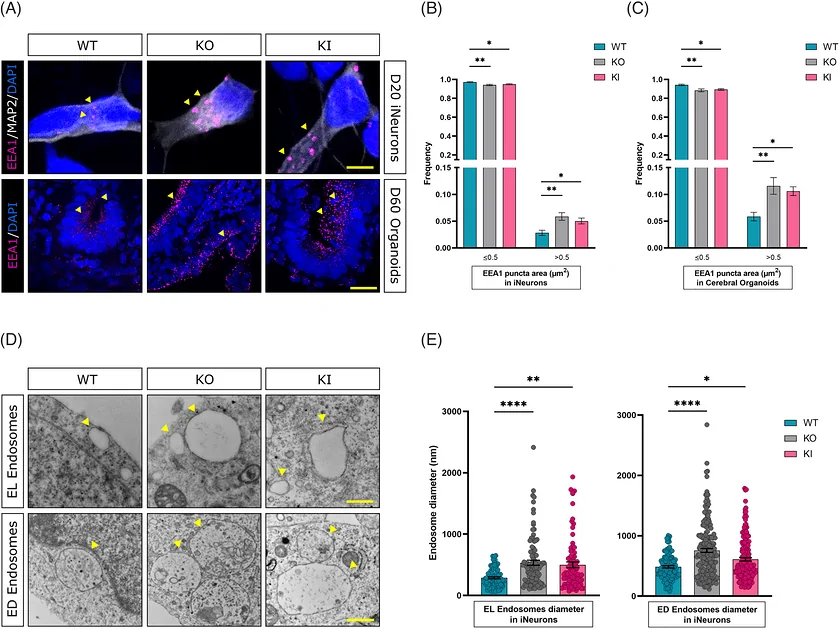
Enlargement and altered distribution of early endosomes in *SORL1* p.Y1816C and KO neurons and organoids. (A) Representative immunofluorescence images showing EEA1‐positive early endosomes in WT, SORLA KO, and KI 2D neurons at day 20 and 3D cerebral organoids at day 60; (B) Size distribution of EEA1+ puncta in 2D neurons, showing the proportion of standard‐sized (≤0.5 µm^2^) and enlarged (> 0.5 µm^2^) EEA1‐positive endosomes across genotypes. *n* = 28‐33 images per genotype (5‐10 cells per image); (C) Size distribution of EEA1+ puncta in 3D cerebral organoids, showing the proportion of standard‐sized (≤0.5 µm^2^) and enlarged (> 0.5 µm^2^) EEA1‐positive endosomes across genotypes. *n* = 5‐7 images per genotype (80‐100 cells per image); (D) Representative TEMs of EL and ED endosomes in WT, KI, and KO neurons; (E) Quantification of the diameter of EL and ED endosomes measured from TEM images. Each dot represents one endosome (biological replicates: n ≥ 3). Data are presented as mean ± SEM. Statistical significance was assessed using Kruskal–Wallis followed by Dunn's multiple comparison test; ****p < 0.001, **p < 0.01, *p < 0.05. Immunofluorescence scale bar: 5 µm (iNs) 5 µm (COs). TEM scale bar: 500 nm. A list of all experimental samples, including iPSC clones used for individual analyses and the number of biological replicates, is provided in Table S2. ED, electron‐dense; EL, electron‐lucent; KI, knock‐in; KO, knock‐out; TEM, transmission electron micrograph; WT, wild‐type.

To evaluate this, we selected Day 20 (D20) for 2D neuronal cultures and day 60 (D60) for 3D cerebral organoids as representative time points for our confocal microscopy analysis. We first performed Immunofluorescence staining for EEA1, a marker of early endosomes, in both models. Representative images from confocal microscopy (Figure 3A) confirmed the previously observed phenotype and revealed visibly enlarged EEA1‐positive endosomes in both SORLA KI and KO neurons compared to WT controls. A similar pattern was observed in 3D cerebral organoids, where KI and KO lines exhibited a greater proportion of visibly enlarged endosomes than WT. Quantitative image analysis further confirmed these observations. By classifying EEA1+ puncta as either normal‐sized (< 0.5 µm^2^) or enlarged (≥0.5 µm^2^), our results demonstrated that both mutant neuronal genotypes showed significantly increased frequency of enlarged endosomes (Figure 3B), and a significant increase was also observed in cerebral organoids (Figure 3C). These findings demonstrate that the AD‐associated *SORL1* mutation promotes endosomal enlargement, consistent with previous reports.^14^

Importantly, although the enlargement of endosomes in SORLA KO neurons has been previously described using immunocytochemistry, a detailed ultrastructural analysis by transmission electron microscopy (TEM) has been lacking. We therefore performed TEM on three biological replicates of differentiated neurons at D20 across genotypes to examine the size, content, and distribution of endosomal structures across the analyzed genotypes. Based on existing literature,^38^ we identified and classified ultrastructural features of the endolysosomal system, including endocytosis and clathrin‐coated pits, electron‐lucent (EL) endosomes, electron‐dense (ED) endosomes, and vesicles undergoing exocytosis (Figure S3A). Notably, EL and ED endosomes were present in WT neurons in a range of sizes, with ED endosomes generally being larger, prompting us to analyze their dimensions separately. Results, as depicted on representative TEM micrographs (Figure 3D) and supported by quantitative analysis (Figure 3E), showed substantial differences in the size of both EL and ED endosomes across genotypes, with both EL and ED endosomes being significantly enlarged in KO neurons and KI neurons compared to WT.

Interestingly, beyond size, we also observed spatial clustering of enlarged endosomes in KI and KO neurons. In these cells, multiple enlarged endosomes frequently appeared clustered within the soma, often visible within a single TEM field (Figure 3D and Figure S3B). In contrast, WT neurons displayed less frequent and more evenly distributed endosomes. It is of note that endosome morphology differed subtly between KO and KI, possibly reflecting variant‐dependent changes in endosomal density or cargo composition. Finally, we did not observe any abnormal fusion events between endosomes in any of the genotypes. Taken together, these results show that the *SORL1* p.Y1816C variant causes endosomal enlargement in 2D neurons and 3D cerebral organoids, closely resembling the phenotype observed in SORLA KO models. Furthermore, characterization of the ultrastructure of enlarged endosomes in SORLA mutant human neurons revealed both morphological changes and clustering behavior that may reflect defects in endosomal trafficking and intracellular positioning, processes essential for normal neuronal function.^39^, ^40^

### 3.4 p.Y1816C leads to APP accumulation in early endosomes, increased secretion of Aβ40 and Aβ42, and enhanced Aβ cluster deposition in cerebral organoids

**FIGURE 4:**
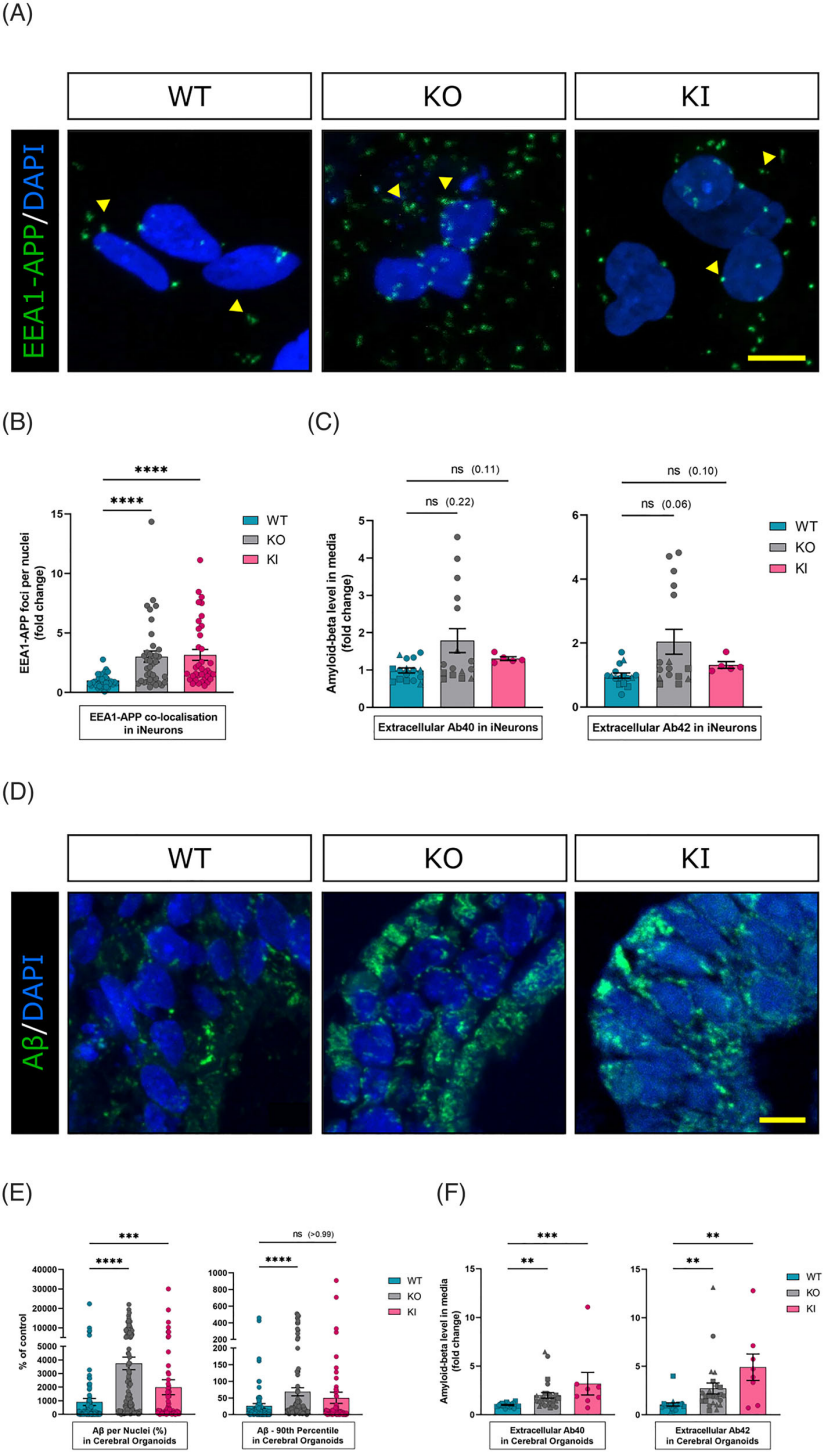
The *SORL1* p.Y1816C mutation promotes APP accumulation in early endosomes and increases Aβ secretion and deposition in neurons and cerebral organoids. (A) Representative images from PLA detecting APP–EEA1 co‐localization in Day 20 2D neurons derived from WT, SORLA KI, and KO iPSC lines. (B) Quantification of PLA signal per nucleus. (C) ELISA measurement of secreted Aβ40 and Aβ42 levels in 2D neuronal culture media. (D) Representative immunofluorescence images of sections from D60 cerebral organoids stained for Aβ. (E) Quantification of Aβ‐positive signal in organoid sections, including: (i) total Aβ‐postive signal per cell (normalized to nuclear volume) and (ii) number of large Aβ‐positive clusters (defined as 90th percentile of particle size). The average signal intensity of the control samples was used as a reference for comparing all analyzed data. (F) ELISA measurements of secreted Aβ40 and Aβ42 to organoid cell culture media. Each dot represents one biological replicate (n ≥ 3). Data represent mean ± SEM. Statistical significance was assessed using Kruskal–Wallis followed by Dunn's multiple comparison test; *p < 0.05, **p < 0.01, ***p < 0.001, ****p < 0.0001. Immunofluorescence scale bar: 10 µm (A) 25 µm (D). A list of all experimental samples, including iPSC clones used for individual analyses and the number of biological replicates, is provided in Table S2. Aβ, amyloid‐β; APP, amyloid precursor protein; ELISA, enzyme‐linked immunosorbent assay; iPSC, induced pluripotent stem cell; KI, knock‐in; KO, knock‐out; PLA, proximity ligation assay; SEM standard error of the mean; WT, wild‐type.

Thus far, we have demonstrated that the *SORL1* p.Y1816C mutation, associated with familial AD, leads to the enlargement of early endosomes in both 2D neurons and 3D cerebral organoids. Importantly, we and others have previously shown that in SORLA‐deficient cellular models or iPSC‐derived neurons, such endosomal enlargement leads to APP accumulation within endosomes and increased cleavage of APP into Aβ40 and Aβ42 peptides.^14^, ^41^, ^42^ However, it remained unclear whether these downstream effects also occur in human neurons carrying a point mutation in *SORL1*, and whether this phenotype is preserved in more complex 3D systems. Thus, to address these questions, we first analyzed 2D neuronal cultures and assessed co‐localization between APP and EEA1 using the PLA. This technique detects protein–protein proximity (≤40 nm), producing a fluorescent signal when two targets, here APP and EEA1, are spatially close, suggesting endosomal co‐localization. Results, as shown in Figure 4A, confirmed that PLA signals were detectable in all tested conditions, but the number of APP–EEA1 foci was markedly elevated in KI and KO neurons compared to WT. Quantitative analysis revealed an approximately three‐fold increase in PLA signal per nucleus in both KI and KO neurons relative to WT (Figure 4B), indicating enhanced APP retention within early endosomes. Additionally, as detected by enzyme‐linked immunosorbent assay (ELISA0, both KI and KO neurons exhibited a trend toward increased Aβ40 and Aβ42 secretion compared to WT (Figure 4C), consistent with prior observations in SORLA‐deficient neurons.^14^, ^42^

Importantly, to determine whether this phenotype extends to 3D models, we next examined Aβ and Aβ40/42 peptides in our cerebral organoids. We previously demonstrated that familial AD mutations in presenilin genes lead to Aβ accumulation in organoids and altered secretion of Aβ peptides.^35^ Applying a similar workflow, we first stained 200‐µm‐thick sections for Aβ and observed increased Aβ signal in both KI and KO organoids compared to WT (Figure 4D). Image analysis quantification further supported these findings. We specifically quantified (i) the overall quantity of Aβ signal per cell (assessed by the volume of Aβ‐positive signal normalized to the volume of cell nuclei per image; Figure 4E), (ii) changes in the overall size of all Aβ‐positive signal particles (visualized as the median volume of Aβ; Figure S4A), and (iii) specifically the number of large Aβ‐positive signal clusters (quantified as the 90^th^ percentile of Aβ particle size; Figure 4E). These analyses revealed that, although the overall size distribution of Aβ particles was not significantly different across genotypes, both KI and KO organoids exhibited a significantly higher amount of Aβ per cell, and a trend toward an increased number of large Aβ‐positive clusters (Figure 4E). Finally, ELISA of organoid‐conditioned media revealed a significant increase in secreted Aβ40 and Aβ42 levels in both KO and KI organoids compared to WT (Figure 4F). Importantly, APP mRNA expression and full‐length APP protein levels remained comparable across WT, KI, and KO lines in both neuronal and organoid models (Figure S4B‐D), indicating that increased Aβ production was not caused by elevated APP expression. Moreover, the APP β‐C terminal fragment (β‐CTF)/full‐length APP ratio was not increased in KI or KO models (Figure S4E,F), suggesting that these phenotypes are not explained by genotype‐dependent accumulation of APP β‐CTFs, but rather reflect altered APP processing within the endosomal system. Together, these results demonstrate that the *SORL1* p.Y1816C variant promotes APP retention in early endosomes, leading to increased Aβ production and accumulation, with 2D neurons and 3D organoids showing reinforcing and complementary data, collectively supporting a shared pathogenic mechanism.

### 3.5 Altered SORLA increases neuritic swelling and deregulates endosomal transport along axons

**FIGURE 5:**
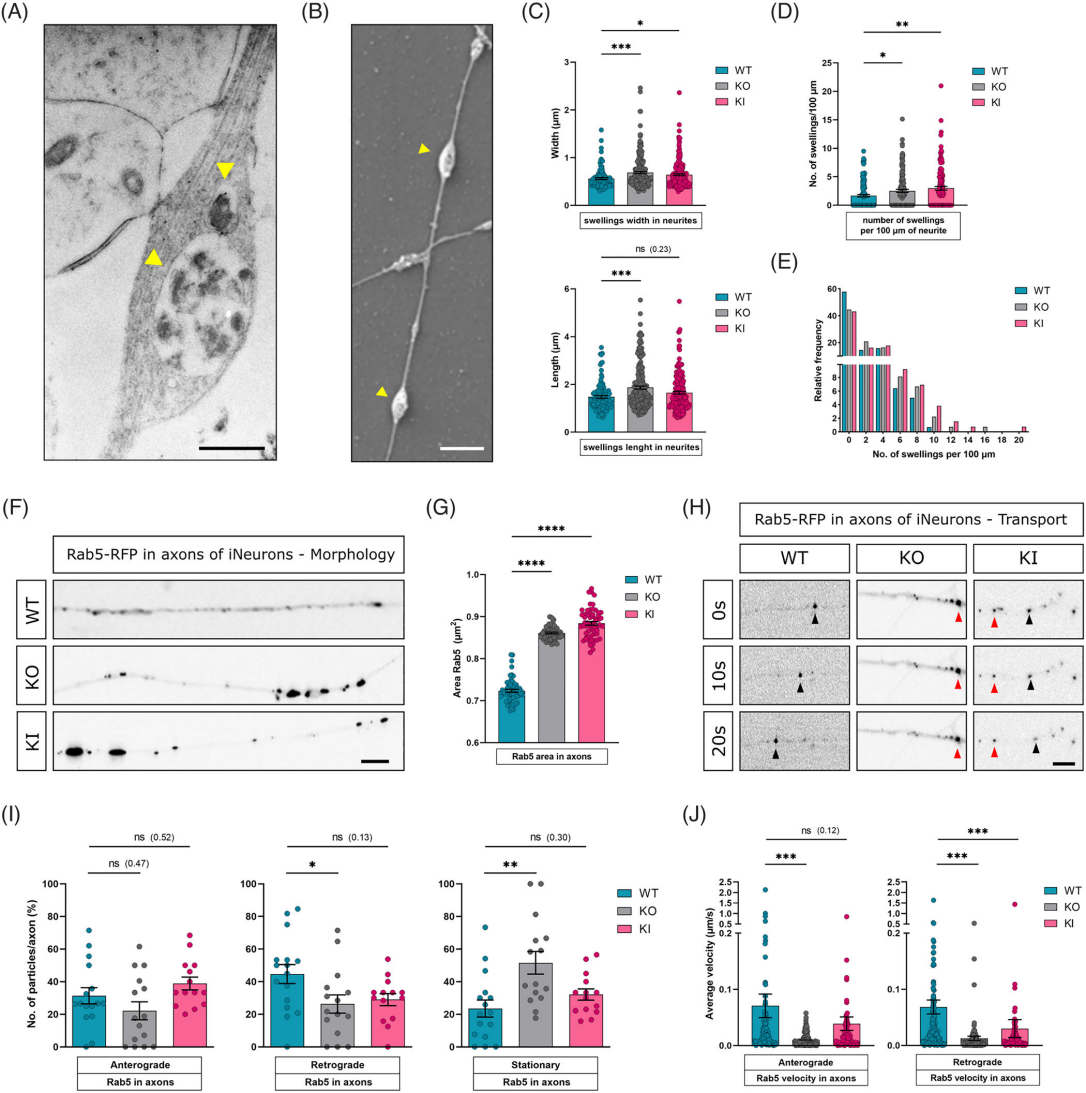
Altered SORLA increases neuritic swelling and deregulates endosomal transport along axons. (A) TEM micrograph showing a cross‐section of a neuritic swelling containing an enlarged endosome. (B) Representative SEM image of differentiated KI neurons displaying neuritic swellings. (C) Quantification of swelling width and length across genotypes. (D) Number of swellings per 100 µm of neurite in WT, KI, and KO neurons. (E) Distribution of the number of swellings per 100 µm segment of neurite. (F) Live‐cell images of Rab5a‐RFP signal in individual axons of WT, KI, and KO neurons at Day 30. (G) Quantification of Rab5+ signal area along axons. (H) Representative pictures showing Rab5+ endosome movement along axons for each genotype. Black arrows point to moving particles, red arrows point to stationary particles. (I) Quantification of axonal transport directionality and proportion of stationary Rab5+ particles. (J) Quantification of the velocity of anterograde and retrograde Rab5+ particle movement over a 60‐second imaging period. In C and D, each dot represents a swelling. A total of 90 projections were analyzed from three biological replicates per genotype; In G, I, and J, each dot represents the mean of Rab5+ endosomes per time point (G), one axon (I), a single endosome (J). All the data were measured in three independent biological replicates. Data represent mean ± SEM. Statistical significance was assessed using one‑way ANOVA followed by Dunnett's Tukey's multiple comparison test or Kruskal–Wallis followed by Dunn's multiple comparison test or t‐test; *p < 0.05, **p < 0.01, ***p < 0.001, ****p < 0.0001. Scale bar on a representative image of axon: 20 µm. TEM scale bar: 500 nm. SEM scale bar: 2 µm. A list of all experimental samples, including iPSC clones used for individual analyses and the number of biological replicates, is provided in Table S2. ANOVA, analysis of variance; iPSC, induced pluripotent stem cell; KI, knock‐in; KO, knock‐out; RFP, red fluorescent protein; SEM, scanning electron microscopy; TEM, transmission electron micrograph; WT, wild‐type.

Thus far, our analyses have focused primarily on the soma of neurons, where we observed endosomal enlargement, defined their ultrastructure via TEM, and demonstrated APP accumulation within these compartments. However, neurons are highly polarized cells, and their proper function depends on efficient intracellular transport not only within the soma but also along axons and dendrites, particularly for the delivery of materials to and from synaptic terminals.^24^ Interestingly, during our TEM analyses of neurons with dysfunctional SORLA, we observed a cross‐section of a neuronal axon exhibiting pronounced swelling, within which we identified an enlarged endosome (Figure 5A). Importantly, axonal swellings are strongly associated with disrupted axonal transport and serve as early indicators of neuronal dysfunction and neurodegeneration in various disease concepts, including AD.^2^, ^43^ Moreover, prior studies on iPSC‐derived AD neurons carrying APP mutations have implicated impaired axonal transport as a central mechanism contributing to disease pathology.^2^, ^23^, ^44^ Thus, this observation prompted us to systematically investigate the effects of SORLA deficiency and the *SORL1* p.Y1816C mutation on axonal morphology and transport dynamics.

We began our analyses by quantifying neuritic swellings in 2D neurons derived from WT, KO, and KI lines. To assess these changes in our model, we performed scanning electron microscopy (SEM) on differentiated WT, KI, and KO neurons using previously established methodology in Pozo *et al.* ^43^ SEM imaging clearly revealed neuritic swellings across all genotypes, allowing for the quantification of multiple morphological parameters (Figure 5B). Our analysis showed that SORLA deficiency and mutation both affect neuritic swelling parameters. Specifically, the width of neuritic swellings was significantly increased in both KO and KI neurons compared to WT (Figure 5C). The length of individual swellings was also significantly longer in KO neurons and showed a trend toward an increase in KI neurons. When we calculated the total number of swellings per 100 µm of neurite, we observed a significantly increased swellings in both KO and KI neurons compared to WT (Figure 5D). Importantly, when we visualized the distribution of swellings per 100 µm, WT neurites generally contained fewer swellings, while KI and KO neurites showed a shift toward a higher frequency of swellings (Figure 5E). Notably, neurites containing more than 10 swellings per 100 µm were rare and exclusively observed in KO and KI neurons, but never in WT. These findings suggest that SORLA dysfunction, either through loss or mutation, leads to structural abnormalities along axons, potentially indicating compromised vesicular transport and endosomal trafficking, which are critical for maintaining neuronal health and polarity.

Thus, we next sought to analyze endosomal trafficking along axons in more detail. To achieve this, we utilized our system of inducible neurons, which were differentiated until D20. Subsequently, we labeled neuronal cultures with CellLight Early Endosomes‐RFP, a BacMam 2.0 reagent that expresses red fluorescent protein (RFP)‐tagged Rab5a, allowing for the live‐cell visualization of early endosomes (hereafter referred to as Rab5+). On D30, we performed live‐cell imaging of individual axons in WT, KI, and KO neurons and quantified their axonal transport dynamics. As shown in Figure 5F and quantified in Figure 5G and Figure S5A, Rab5+ signals already displayed distinct patterns across genotypes. In WT neurons, Rab5+ endosomes appeared as small puncta distributed evenly along the axon. In a strong contrast, KI and especially KO neurons exhibited large Rab5+ clusters, indicating disrupted endosomal distribution. Importantly, changes in the area of Rab5^+^ endosomes were observed only when comparing WT, KO, and KI genotypes, but not across time within the same genotype, suggesting that once endosomes are enlarged, their size remains stable over at least a 60‐second period (Figure S5A).

Interestingly, quantitative analysis of Rab5+ endosomal transport revealed clear genotype‐dependent differences in directionality and motility (Figure 5H,I, and Figure S5B). In WT neurons, Rab5+ particles displayed typical transport behavior, with ∼45% moving retrogradely, 32% anterogradely, and 23% remaining stationary, consistent with previous reports.^19^ In KI neurons carrying the p.Y1816C variant, this distribution was altered, showing a reduced proportion of retrograde transport and a similar proportion of stationary particles, with modest differences in the anterograde fraction. The effect was more pronounced in SORLA KO neurons, where over 50% of Rab5+ particles remained stationary and fewer particles moved in either direction.

We next focused on the dynamic properties of the moving particles over a 60‐second imaging period, excluding particles lower than 0.001 µm/s. Importantly, we found that velocities of both anterograde and retrograde Rab5+ particles were significantly reduced in KO neurons compared to WT, and trended lower in KI neurons (Figure 5J). The total distance traveled by individual endosomes, referred to as track length, was also significantly shorter in both KI and KO neurons (Figure S5C). Taken together, these results indicate that the *SORL1* p.Y1816C mutation, and more severely, complete SORLA loss, compromise both the movement and spatial distribution of Rab5+ endosomes in axons, indicating a key role for SORLA in axonal endosomal trafficking.

Lastly, as an initial exploration of the transport machinery, we analyzed the expression and localization of Dynactin Subunit 1 (DCTN1), a key component of the dynactin complex involved in dynein‐mediated retrograde transport. While total DCTN1 levels were unchanged in whole‐cell lysates from 2D neurons and 3D cerebral organoids, DCTN1 abundance was decreased in endosome‐enriched fractions from *SORL1* KI and KO models (Figure S5D,E). Although preliminary and requiring further validation, these data suggest an altered association of dynactin‐dependent transport machinery with endosomal compartments in SORL1 KI and KO models, potentially contributing to impaired retrograde endosomal transport.

### 3.6 SORLA alterations affect the axonal transport of APP

**FIGURE 6:**
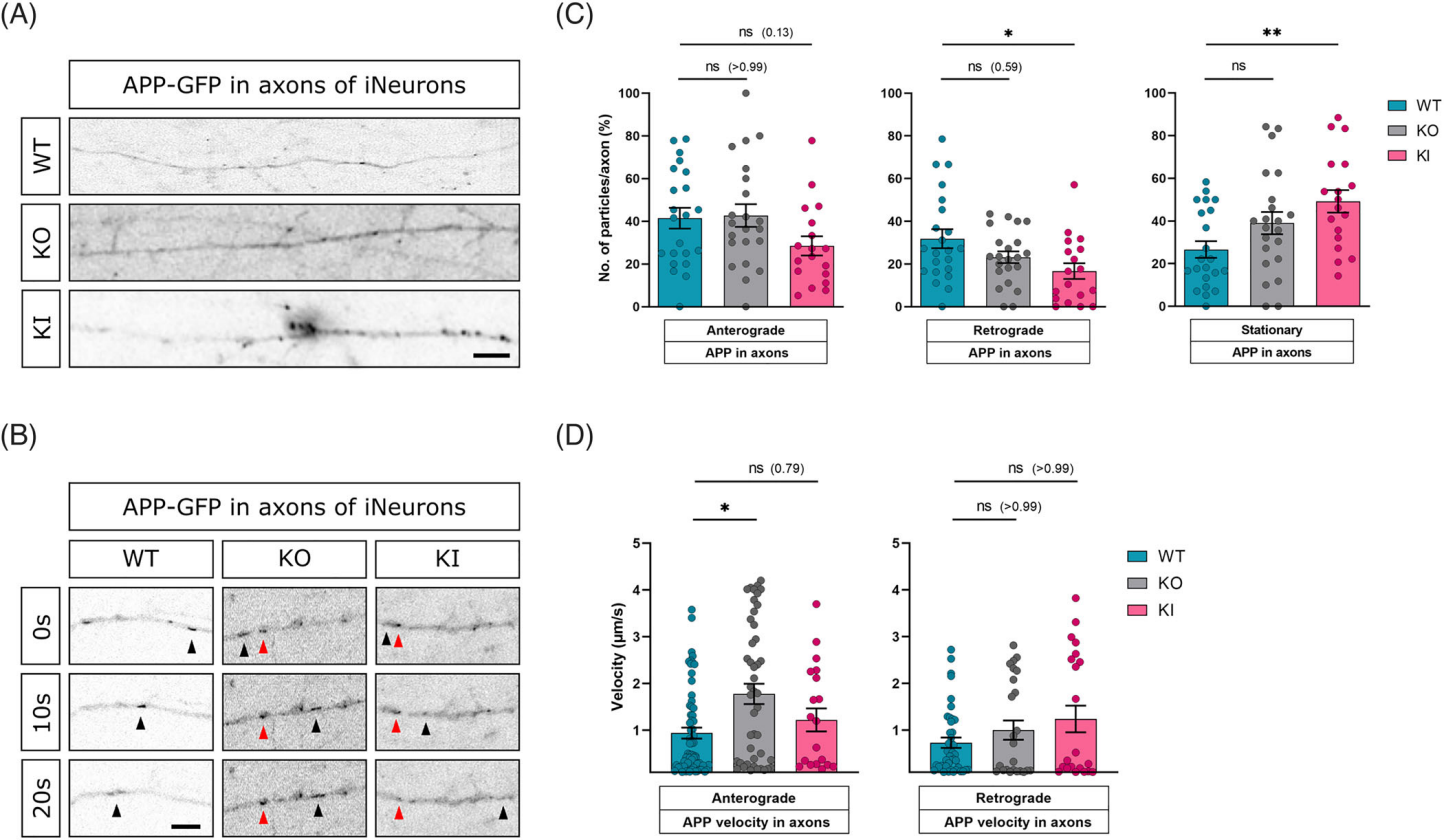
SORLA alterations impair axonal transport of APP in human neurons. (A) Representative still frames from live imaging of 2D neurons expressing APP–GFP, showing axonal localization of GFP signal in WT, KI, and KO neurons; (B) Representative pictures illustrating APP–GFP particle movement along axons in WT, KI, and KO neurons. Black arrows point to moving particles, Red arrows point to stationary particles; (C) Quantification of APP–GFP transport parameters showing the proportion of particles moving anterogradely, retrogradely, or remaining stationary; (D) Velocity of fast‐moving APP–GFP particles (≥0.1 µm/s) in anterograde and retrograde directions across genotypes. Each dot represents one axon (C) or a moving APP particle (D). Data were measured in three independent biological replicates. Data represent mean ± SEM. Statistical significance was assessed using one‑way ANOVA followed by Dunnett's Tukey's multiple comparison test, Kruskal–Wallis followed by Dunn's multiple comparison test or t‐test; *p < 0.05, **p < 0.01. Scale bar on a representative image of an axon: 20 µm. A list of all experimental samples, including iPSC clones used for individual analyses and the number of biological replicates, is provided in Table S2. ANOVA, analysis of variance; APP, amyloid precursor protein; GFP, green fluorescent protein; iPSC, induced pluripotent stem cell; KI, knock‐in; KO, knock‐out; SEM, scanning electron microscopy; WT, wild‐type.

Given the striking disruption of endosomal distribution and trafficking along axons in our previous experiments, we next investigated whether axonal transport of APP, a critical protein in AD pathology, is similarly affected. Earlier studies, including ours, have demonstrated that familial AD mutations, such as the Swedish APP variant, impair retrograde motor function and broadly disturb axonal transport dynamics.^23^ However, whether the SORLA protein has any effect on the axonal transport of APP remained unaddressed. Thus, to assess APP trafficking in this context, we used lentiviral constructs expressing GFP‐tagged wild‐type APP (APP–GFP; previously used in^23^ as APPwt–GFP) and performed live imaging of axonal transport in 2D inducible neurons derived from WT, KO, and KI SORLA lines. As shown in Figure 6A, in contrast to the clustered Rab5+ signal observed previously, the APP–GFP signal appeared more uniformly distributed along axons.

However, tracking analyses revealed significant alterations in APP transport dynamics. Both *SORL1* KO and KI neurons showed a marked increase in the proportion of stationary APP–GFP particles compared to WT neurons. Although anterograde and retrograde transport remained detectable and followed a similar directional pattern across genotypes, the overall number of moving APP particles was reduced in KO and KI neurons, indicating a shift toward particle stalling. Representative images are shown in Figure 6B, with quantification in Figure 6C and Figure S6A. Moreover, the track length, which represents the total distance traveled by individual APP–GFP particles, was significantly shorter in KO and KI neurons compared to WT, suggesting impaired transport range and reduced mobility within axons (Figure S6B). Additionally, analysis of the fast‐moving subset of APP–GFP particles (≥0.1 µm/s) revealed that both anterograde and retrograde velocities were significantly elevated or showed an upward trend in KO and KI neurons compared to WT (Figure 6D). In line with this impaired APP trafficking, preliminary analysis of co‐localization in MAP2‐positive neurites showed a trend toward increased localization of both full‐length APP and Aβ within EEA1‐positive endosomes in SORL1 KI and KO neurons (Figure S6C). These findings suggest that SORLA dysfunction not only increases particle stalling but also alters the kinetics of moving APP vesicles, potentially reflecting compensatory changes in motor‐cargo dynamics.

Finally, to test whether this APP transport phenotype depends on amyloidogenic processing, we treated WT and SORL1 KO neurons with a BACE1 inhibitor. Although BACE1 inhibition successfully reduced secreted Aβ40 and Aβ42 and increased full‐length APP levels, it did not rescue the altered distribution of stationary versus moving APP–GFP particles in SORL1 KO neurons (Figure S6D‐F). Together with the observed impairments in Rab5^+^ endosomal transport, our results highlight a broader and previously underappreciated role for SORLA in regulating axonal transport and maintaining neuronal homeostasis in the context of AD pathology.

### 3.7 Electrophysiological measurements show hyperexcitable neurons with SORLA KO and SORL1 p.Y1816C variant

**FIGURE 7:**
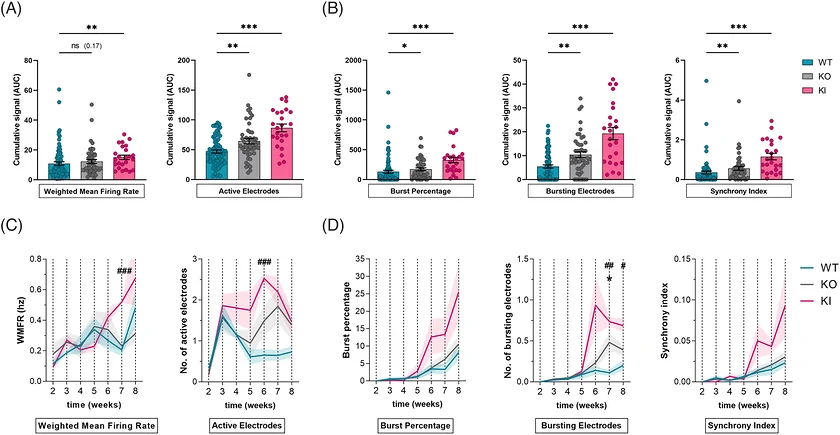
Electrophysiological analysis reveals increased neuronal excitability in SORLA KO and SORLA p.Y1816C mutant neurons; (A) Quantification of individual activity parameters recorded by MEA: weighted mean firing rate and number of active electrodes. (B) Quantification of network‐level parameters: burst percentage, number of bursting electrodes, and synchrony index. (C) Time‐course visualization of weighted mean firing rate and number of active electrodes over several weeks of neuronal differentiation. (D) Time‐course analysis of burst percentage, bursting electrodes, and synchrony index across the same period. Each data point represents one well of neuronal culture derived from a different iPSC line. 48, 48, and 24 datapoints are shown from WT, KO, and KI neurons, respectively. Data represent mean ± SEM; Statistical significance was determined using Kruskal–Wallis followed by Dunn's multiple comparison test or Two‐way ANOVA followed by Dunnett's post‐test; *p < 0.05, **p < 0.01, ***p < 0.001. WT vs KO = *; WT vs KI = #. A list of all experimental samples, including iPSC clones used for individual analyses and the number of biological replicates, is provided in Table S2. ANOVA, analysis of variance; iPSC, induced pluripotent stem cell; KI, knock‐in; KO, knock‐out; MEA, microelectrode array; SEM standard error of the mean; WT, wild‐type.

Finally, to investigate the functional consequences of SORLA deficiency and the *SORL1* p.Y1816C mutation on neuronal activity, we performed long‐term electrophysiological recordings using MEA technology on 2D differentiated neurons from D14 to D56. We assessed both individual electrode and global network parameters across WT, KO, and KI neuronal cultures. For individual electrode activity (Figure 7A), we analyzed the weighted mean firing rate, which showed a significant increase in KI neurons and a strong upward trend in KO neurons compared to WT. Similarly, the number of active electrodes was significantly elevated in both KI and KO neurons, suggesting increased spontaneous activity. Next, we evaluated network‐level properties (Figure 7B). The burst percentage, a measure of neuronal firing maturation, was significantly higher in both KI and KO neurons, and we also found a significant increase in the number of bursting electrodes, indicating a maturation of electrophysiological properties of the neuronal cultures. Finally, the synchrony index, reflecting the degree of temporal coordination between electrodes and network engagement, was also significantly elevated in both mutant conditions.

To provide molecular context for these functional changes, we also analyzed selected pre‐ and postsynaptic markers at Day 30. Western blot analysis showed genotype‐dependent changes in the presynaptic markers SYN1 and SYP and the postsynaptic markers PSD95 and HOMER1, suggesting that altered synaptic protein composition may contribute to the observed network phenotype (Figure S7).

Lastly, to explore when these functional changes emerged, we visualized the time course of neuronal maturation on MEA over several weeks. Across multiple measurements, most electrophysiological differences became visible, and often statistically significant, around 6 weeks of differentiation, highlighting a delayed onset of network dysfunction. The longitudinal evolution of each parameter is presented in Figures 7C and 7D. Taken together, these results strongly indicate that both SORLA loss and the AD‐causing mutation in *SORL1* p.Y1816C lead to increased neuronal excitability and altered network dynamics in human neurons.

## 4 DISCUSSION

In this study, we examined the pathogenic impact of the Alzheimer's‐associated *SORL1* p.Y1816C variant using complementary human iPSC‐derived models: 2D neurons and 3D cerebral organoids. The variant impaired SORLA maturation and shedding without affecting differentiation. Both the KO and KI models showed marked early‐endosome enlargement in neurons and organoids, accompanied by increased APP retention, elevated Aβ40/42 production, and Aβ deposition. We further identified pronounced neuritic swellings and major defects in axonal transport of Rab5+ endosomes and APP, including increased stalling and reduced velocities. Finally, SORLA dysfunction resulted in hyperactive neuronal networks with increased firing, burst activity, and synchrony. Together, these findings demonstrate that SORLA loss or mutation disrupts endosomal trafficking and APP processing, leading to neuronal dysfunction relevant to Alzheimer's disease.

### 4.1 Altered biology of SORLA

At the beginning of our study, we conducted a series of experiments to investigate key aspects of SORLA protein biology, specifically its maturation and shedding.^36^ Notably, previous studies by us and others have shown that these processes are frequently disrupted in pathogenic *SORL1* variants associated with AD,^14^, ^16^, ^20^, ^42^ leading to protein retention in the endoplasmic reticulum and loss of downstream trafficking functions.^45^ Additional studies showed that reduced SORLA maturation disrupts its interaction with the retromer complex and impairs APP sorting, thereby contributing to increased amyloidogenic processing.^10^, ^21^, ^46^ Importantly, once trafficked to the cell surface, SORLA undergoes proteolytic cleavage by ADAM family proteases, releasing its soluble ectodomain (sSORLA), an indicator of proper surface trafficking. We and others have previously demonstrated that sSORLA shedding is reduced when the receptor is dysfunctional or retained in intracellular compartments.^20^ Notably, sSORLA levels were also decreased in cerebrospinal fluid of *SORL1* mutation carriers.^37^ Our current findings expand this understanding by showing that the SORL1 p.Y1816C variant impairs maturation and reduces SORLA shedding in human‐relevant models with endogenous *SORL1* expression, providing further functional evidence that this missense variant is pathogenic.

### 4.2 Endosomal enlargement and APP processing

Given the central role of SORLA in endosomal protein sorting, we next examined how its dysfunction impacts endosomal architecture and APP metabolism. We first focused on endosomal enlargement, a cellular pathology increasingly recognized as a general upstream mechanism in AD pathogenesis^1^, ^3^ as disruption of endosomal function may impair trafficking of key neuronal cargo, including APP, neurotrophic receptors, and lipids, thereby triggering downstream neurodegenerative cascades.^3^, ^6^, ^9^ Indeed, endosomal enlargement has been extensively reported in models of *SORL1* deficiency or mutants,^14^, ^16^, ^18^, ^19^, ^45^ as well as in other AD‐related genes such as *PSEN1*, *APP*, and *APOE4*.^7^, ^9^, ^17^, ^47^ Notably, this phenotype is especially relevant to APP processing, as BACE1, the enzyme initiating the amyloidogenic cleavage pathway, predominantly acts within early endosomes.^48^, ^49^, ^50^ Thus, retention of APP in enlarged endosomes increases its accessibility to BACE1, promoting the generation and secretion of Aβ40 and Aβ42 peptides, a central feature of AD pathology. Importantly, previous studies using *SORL1* KO models, overexpression systems, and iPSC‐derived neurons have confirmed that SORLA loss results in increased APP accumulation in endosomes and elevated Aβ secretion.^10^, ^13^, ^14^ Consistent with these findings, our data now show that the *SORL1* p.Y1816C mutation leads to a significant enlargement of early endosomes, increased APP retention within these compartments, and enhanced production of Aβ40 and Aβ42 peptides. Notably, we provide the first ultrastructural evidence of early endosome enlargement in human neurons using TEM, offering direct visual confirmation of altered endosomal architecture. Moreover, we demonstrate that these phenotypes are faithfully recapitulated in 3D cerebral organoids, confirming that SORLA‐related endosomal dysfunction is preserved in more complex, tissue‐like models of the human brain.

Notably, previous evidence indicates that endosomal enlargement can be independently triggered by APP β‐CTFs.^17^, ^51^, ^52^, ^53^ Consistent with this, BACE1 inhibition rescued endosomal swelling in a large cohort of familial AD hiPSC‐derived neurons.^17^ However, prior work by Knupp et al.^14^ in *SORL1*‐deficient neurons demonstrated that BACE1 inhibition reduced Aβ levels but did not rescue endosomal swelling in *SORL1* knockout hiPSC‐derived neurons, indicating that this phenotype can occur independently of amyloidogenic APP processing and is directly tied to the loss of an endosomal sorting receptor. These findings support the interpretation that SORLA dysfunction disrupts endosomal homeostasis through broader trafficking defects rather than solely through β‐CTF–dependent pathways.

### 4.3 Compromised axonal biology and axonal transport in AD

While somatic vesicle trafficking has been the primary focus of many studies on SORLA function, far less is known about its potential roles in the axonal compartment. Indeed, neurons rely on precisely regulated long‐range intracellular transport between the cell body and distant axonal or dendritic compartments, powered by motor proteins, dyneins and kinesins.^24^ Interestingly, among the cargoes trafficked along axons, Rab5‐positive early endosomes play a significant role, carrying molecules such as APP, TrkB, and recycling receptors in both anterograde and retrograde directions.^54^, ^55^ Disruption of this transport system has been shown in AD models carrying the APP Swedish mutation, where endosomes and APP particles exhibit abnormal stalling and impaired motility.^23^ Additionally, one well‐documented hallmark of compromised axonal integrity is the formation of neuritic or axonal swellings, focal enlargements associated with vesicular accumulation and impaired transport,^2^, ^26^, ^43^ observed across a range of neurodegenerative diseases, including AD (reviewed in Ref.^25^). Our current findings now extend this knowledge by revealing that both *SORL1* p.Y1816C mutant and SORLA KO neurons exhibit clear morphological and functional signs of axonal dysfunction. We observed pronounced neuritic swellings, often containing enlarged endosomes. Live‐cell imaging confirmed that Rab5+ endosomes in SORLA‐defective neurons exhibit abnormal clustering, increased stalling, and reduced bidirectional transport velocities. Parallel analyses of APP trafficking revealed similar impairments, including an increased proportion of stationary APP–GFP particles, further supporting a direct link between SORLA dysfunction and axonal pathology.

Additionally, consistent with this impaired APP trafficking, our preliminary data further suggested increased localization of full‐length APP and Aβ within endosomes in neurites of SORL1 KI and KO neurons. This supports the possibility that SORLA dysfunction promotes local retention of APP‐related cargo in early endosomal compartments, forming local sites of amyloidogenic processing within neurites. Importantly, however, BACE1 inhibition reduced Aβ40 and Aβ42 secretion but did not rescue the altered distribution of stationary and moving APP–GFP particles in SORL1 KO neurons. Together with the unchanged APP β‐CTF/full‐length APP ratios in our SORL1 KO and KI models, these findings suggest that the axonal transport defect is not primarily driven by increased Aβ production or β‐CTF accumulation but rather reflects a broader disruption of SORLA‐dependent endosomal trafficking. Mechanistically, our analysis of the dynactin motor protein provides an initial indication that SORLA dysfunction may alter the association of transport machinery with endosomal compartments. Taken together, our results demonstrate that SORLA is essential not only for somatic vesicle trafficking but also for maintaining axonal homeostasis and transport dynamics, thereby implicating *SORL1* dysfunction as a mechanistic driver of axonal pathology in AD.

### 4.4 Hyperexcitability and AD

Multiple studies thus far have demonstrated that neurons carrying AD‐causing mutations exhibit aberrant network activity and increased excitability, including studies on human iPSC‐derived neurons and organoids with familial AD mutations^17^, ^28^ or mouse models of AD showing spontaneous epileptiform activity.^29^, ^30^ Clinical observations also support this, as increased subclinical seizure activity and cortical hyperexcitability have been detected in individuals with early‐onset or autosomal dominant AD, even in the preclinical stages.^56^, ^57^ Notably, a study by Zhou et al. reported that iPSC‐derived neurons carrying the APP Swedish mutation display increased spontaneous synaptic activity, which they attributed to the action of Aβ peptides accumulating in the culture media, acting in a paracrine manner to modulate synaptic excitability.^31^ However, despite SORLA being known to regulate APP trafficking and Aβ generation, its role in modulating the electrophysiological properties of neurons has remained largely unexplored. Only one prior study by the Young lab reported increased excitability in SORL1 KO human neurons.^18^ Our data now demonstrate that both *SORL1* p.Y1816C and SORLA KO neurons exhibit deregulated electrophysiology. This included elevated firing rates, a greater number of active and bursting electrodes, and increased synchrony across the network. Future studies including astrocytes will be valuable to determine how glial regulation of synaptic activity may influence SORL1‐dependent network phenotypes.

Importantly, these functional findings are further corroborated by Williams *et al.* (submitted back‐to‐back with this manuscript), who not only observed hyperexcitability in *SORL1*‐deficient neurons, confirming our findings, but also describe that this hyperexcitability is not solely due to the overproduction of Aβ but rather due to the mis‐trafficking of synaptic proteins. Together, our findings and those of Williams *et al.* add compelling evidence that *SORL1* dysfunction disrupts multiple layers of neuronal biology, ranging from subcellular trafficking to synaptic function, reinforcing the emerging view that endosomal recycling defects are central, upstream drivers in AD pathogenesis.

## CONFLICT OF INTEREST STATEMENT

The authors declare no conflicts of interest. Author disclosures are available in the Supporting Information.

## CONSENT STATEMENT

Informed consent from patients was not necessary.

## Supporting information



Supporting Information

Supporting Information

Supporting Information

Supporting Information
