# Supplementary material for: SORL1 variant links endosomal amyloid precursor protein mis‐sorting to axonal transport defects and neuronal dysfunction
Source: Alzheimers Dement. 2026 Aug 22;22(8):e71782. doi: 10.1002/alz.71782 (PMC13498896; doi:10.1002/alz.71782)
Supplement: Supplementary file 2 [file ALZ-22-e71782-s001.docx]

**Supplementary Tables**

**Table 1: Cell lines used**

| **cell line name in the manuscript** | **genotype** | **publication about derivation and characterization** | **schematic representation in the manuscript** |
| --- | --- | --- | --- |
| i3N iPSCs WT parental cell line | Wild-type (WT; apparently healthy control) | Fernandopulle et al., 2018 | ● |
| i3N iPSCs MOCK Clone C3 | Wild-type (WT; apparently healthy control) | This manuscript | ■ |
| i3N iPSCs MOCK Clone C11 | Wild-type (WT; apparently healthy control) | This manuscript | ▲ |
| i3N iPSCs *SORL1* KO Clone D107 | *SORL1* Knock Out (KO) | Jensen et al., 2024 | ● |
| i3N iPSCs *SORL1* KO Clone K4 | *SORL1* Knock Out (KO) | This manuscript | ■ |
| i3N iPSCs *SORL1* KO Clone K7 | *SORL1* Knock Out (KO) | This manuscript | ▲ |
| i3N iPSCs *SORL1* p.Y1816C KI Clone F37 | *SORL1* p.Y1816C Knock In (KI) | Jensen et al., 2024 | ● |

**Table 2: Number of replicates per experiment**

| **figure** | **experimental method** | | **used cell line, condition** | **total No. of biological replicates from all cell lines (n)** | **total No. of individual samples** | **sample type** | **number of samples per timepoint (shown in the figure)** | | | | | |
| --- | --- | --- | --- | --- | --- | --- | --- | --- | --- | --- | --- | --- |
|  |  |  |  |  |  |  | **D0** | | | | | |
| 1C-D | WB | | i3N iPSCs WT parental cell line | 4 | 4 | iPSCs; cell lysate | 4 | | | | | |
|  |  |  | i3N iPSCs MOCK Clone C3 | 3 | 3 | iPSCs; cell lysate | 3 | | | | | |
|  |  |  | i3N iPSCs MOCK Clone C11 | 3 | 3 | iPSCs; cell lysate | 3 | | | | | |
|  |  |  | i3N iPSCs *SORL1* KO Clone D107 | 4 | 4 | iPSCs; cell lysate | 4 | | | | | |
|  |  |  | i3N iPSCs *SORL1* KO Clone K4 | 3 | 3 | iPSCs; cell lysate | 3 | | | | | |
|  |  |  | i3N iPSCs *SORL1* KO Clone K7 | 3 | 3 | iPSCs; cell lysate | 3 | | | | | |
|  |  |  | i3N iPSCs *SORL1* p.Y1816C KI Clone F37 | 4 | 4 | iPSCs; cell lysate | 4 | | | | | |
| 1E-F | WB | | i3N iPSCs WT parental cell line | 5 | 5 | iPSCs; cell lysate | 5 | | | | | |
|  |  |  | i3N iPSCs MOCK Clone C3 | 2 | 2 | iPSCs; cell lysate | 2 | | | | | |
|  |  |  | i3N iPSCs MOCK Clone C11 | 2 | 2 | iPSCs; cell lysate | 2 | | | | | |
|  |  |  | i3N iPSCs *SORL1* p.Y1816C KI Clone F37 | 5 | 5 | iPSCs; cell lysate | 5 | | | | | |
| S1B | PCR/ELFO | | i3N iPSCs WT parental cell line | 1 | 1 | iPSCs; DNA | 1 | | | | | |
|  |  |  | i3N iPSCs *SORL1* KO Clone D107 | 1 | 1 | iPSCs; DNA | 1 | | | | | |
|  |  |  | i3N iPSCs *SORL1* KO Clone K4 | 1 | 1 | iPSCs; DNA | 1 | | | | | |
|  |  |  | i3N iPSCs *SORL1* KO Clone K7 | 1 | 1 | iPSCs; DNA | 1 | | | | | |
| S1D | PCR | | i3N iPSCs WT parental cell line | 2 | 2 | iPSCs; DNA | 2 | | | | | |
| S1F | RT-PCR/ELFO | | i3N iPSCs WT parental cell line | 1 | 1 | iPSCs; RNA | 1 | | | | | |
|  |  |  | i3N iPSCs *SORL1* p.Y1816C KI Clone F37 | 1 | 1 | iPSCs; RNA | 1 | | | | | |
|  | | | | | | | **D20** | | **D30** | | **D40** | |
| 2C; S2A; S2A’; S4B | qPCR | | i3N iPSCs WT parental cell line | 3 | 9 | iNeurons; RNA | 3 | | 3 | | 3 | |
|  |  |  | i3N iPSCs MOCK Clone C3 | 3 | 9 | iNeurons; RNA | 3 | | 3 | | 3 | |
|  |  |  | i3N iPSCs MOCK Clone C11 | 3 | 9 | iNeurons; RNA | 3 | | 3 | | 3 | |
|  |  |  | i3N iPSCs SORL1 KO Clone D107 | 3 | 9 | iNeurons; RNA | 3 | | 3 | | 3 | |
|  |  |  | i3N iPSCs SORL1 KO Clone K4 | 3 | 9 | iNeurons; RNA | 3 | | 3 | | 3 | |
|  |  |  | i3N iPSCs SORL1 KO Clone K7 | 3 | 9 | iNeurons; RNA | 3 | | 3 | | 3 | |
|  |  |  | i3N iPSCs SORL1 p.Y1816C KI Clone F37 | 3 | 12 | iNeurons; RNA | 4 | | 4 | | 4 | |
| 2D | WB | | i3N iPSCs WT parental cell line | 4 | 12 | iNeurons; cell lysate | 4 | | 4 | | 4 | |
|  |  |  | i3N iPSCs MOCK Clone C3 | 4 | 12 | iNeurons; cell lysate | 4 | | 4 | | 4 | |
|  |  |  | i3N iPSCs MOCK Clone C11 | 4 | 12 | iNeurons; cell lysate | 4 | | 4 | | 4 | |
|  |  |  | i3N iPSCs SORL1 p.Y1816C KI Clone F37 | 3 | 12 | iNeurons; cell lysate | 4 | | 4 | | 4 | |
| S2B; S4D | WB | | i3N iPSCs WT parental cell line | 3 | 9 | iNeurons; cell lysate | 3 | | 3 | | 3 | |
|  |  |  | i3N iPSCs MOCK Clone C3 | 3 | 9 | iNeurons; cell lysate | 3 | | 3 | | 3 | |
|  |  |  | i3N iPSCs MOCK Clone C11 | 3 | 9 | iNeurons; cell lysate | 3 | | 3 | | 3 | |
|  |  |  | i3N iPSCs SORL1 KO Clone D107 | 3 | 9 | iNeurons; cell lysate | 3 | | 3 | | 3 | |
|  |  |  | i3N iPSCs SORL1 KO Clone K4 | 3 | 9 | iNeurons; cell lysate | 3 | | 3 | | 3 | |
|  |  |  | i3N iPSCs SORL1 KO Clone K7 | 3 | 9 | iNeurons; cell lysate | 3 | | 3 | | 3 | |
|  |  |  | i3N iPSCs SORL1 p.Y1816C KI Clone F37 | 3 | 9 | iNeurons; cell lysate | 3 | | 3 | | 3 | |
| 2E | ELISA | | i3N iPSCs WT parental cell line | 4 | 5 | iNeurons; cell media |  | | 5 | |  | |
|  |  |  | i3N iPSCs MOCK Clone C3 | 3 | 4 | iNeurons; cell media |  | | 4 | |  | |
|  |  |  | i3N iPSCs MOCK Clone C11 | 4 | 4 | iNeurons; cell media |  | | 4 | |  | |
|  |  |  | i3N iPSCs SORL1 p.Y1816C KI Clone F37 | 4 | 6 | iNeurons; cell media |  | | 6 | |  | |
|  | | | | | | | **D9** | **D15** | | **D30** | | **D60** |
| 2H; S2C; S2C’; S4B | qPCR | | i3N iPSCs WT parental cell line | 3 | 12 | Organoids; RNA | 3 | 3 | | 3 | | 3 |
|  |  |  | i3N iPSCs MOCK Clone C3 | 3 | 12 | Organoids; RNA | 3 | 3 | | 3 | | 3 |
|  |  |  | i3N iPSCs MOCK Clone C11 | 3 | 12 | Organoids; RNA | 3 | 3 | | 3 | | 3 |
|  |  |  | i3N iPSCs SORL1 KO Clone D107 | 3 | 12 | Organoids; RNA | 3 | 3 | | 3 | | 3 |
|  |  |  | i3N iPSCs SORL1 KO Clone K4 | 3 | 12 | Organoids; RNA | 3 | 3 | | 3 | | 3 |
|  |  |  | i3N iPSCs SORL1 KO Clone K7 | 3 | 12 | Organoids; RNA | 3 | 3 | | 3 | | 3 |
|  |  |  | i3N iPSCs SORL1 p.Y1816C KI Clone F37 | 3 | 12 | Organoids; RNA | 3 | 3 | | 3 | | 3 |
| 2I; S2D; S4E | WB | | i3N iPSCs WT parental cell line | 3 | 12 | Organoids; cell lysate | 3 | 3 | | 3 | | 3 |
|  |  |  | i3N iPSCs MOCK Clone C3 | 3 | 12 | Organoids; cell lysate | 3 | 3 | | 3 | | 3 |
|  |  |  | i3N iPSCs MOCK Clone C11 | 3 | 12 | Organoids; cell lysate | 3 | 3 | | 3 | | 3 |
|  |  |  | i3N iPSCs SORL1 KO Clone D107 | 3 | 12 | Organoids; cell lysate | 3 | 3 | | 3 | | 3 |
|  |  |  | i3N iPSCs SORL1 KO Clone K4 | 3 | 12 | Organoids; cell lysate | 3 | 3 | | 3 | | 3 |
|  |  |  | i3N iPSCs SORL1 KO Clone K7 | 3 | 12 | Organoids; cell lysate | 3 | 3 | | 3 | | 3 |
|  |  |  | i3N iPSCs SORL1 p.Y1816C KI Clone F37 | 3 | 12 | Organoids; cell lysate | 3 | 3 | | 3 | | 3 |
| 2J | ELISA | | i3N iPSCs WT parental cell line | 5 | 5 | Organoids; media |  |  | |  | | 5 |
|  |  |  | i3N iPSCs MOCK Clone C3 | 4 | 4 | Organoids; media |  |  | |  | | 4 |
|  |  |  | i3N iPSCs MOCK Clone C11 | 4 | 4 | Organoids; media |  |  | |  | | 4 |
|  |  |  | i3N iPSCs SORL1 p.Y1816C KI Clone F37 | 5 | 5 | Organoids; media |  |  | |  | | 5 |
| S2E-F | WB | | i3N iPSCs WT parental cell line | 2 | 2 | Organoids; media |  |  | |  | | 2 |
|  |  |  | i3N iPSCs SORL1 KO Clone D107 | 2 | 2 | Organoids; media |  |  | |  | | 2 |
|  |  |  | i3N iPSCs SORL1 p.Y1816C KI Clone F37 | 2 | 2 | Organoids; media |  |  | |  | | 2 |
|  | | | | | | | **D20** | | **D30** | | **D40** | |
| 3A,B; S3A | | IF | i3N iPSCs WT parental cell line | 3 | 3 | iNeurons; coverslips |  | | 3 | |  | |
|  |  |  | i3N iPSCs MOCK Clone C3 | 3 | 3 | iNeurons; coverslips |  | | 3 | |  | |
|  |  |  | i3N iPSCs MOCK Clone C11 | 2 | 2 | iNeurons; coverslips |  | | 2 | |  | |
|  |  |  | i3N iPSCs SORL1 KO Clone D107 | 4 | 4 | iNeurons; coverslips |  | | 4 | |  | |
|  |  |  | i3N iPSCs SORL1 KO Clone K4 | 2 | 2 | iNeurons; coverslips |  | | 2 | |  | |
|  |  |  | i3N iPSCs SORL1 KO Clone K7 | 2 | 2 | iNeurons; coverslips |  | | 2 | |  | |
|  |  |  | i3N iPSCs SORL1 p.Y1816C KI Clone F37 | 3 | 3 | iNeurons; coverslips |  | | 3 | |  | |
|  | | | | | | | **D9** | **D15** | | **D30** | | **D60** |
| 3A,C; S3A | IF | | i3N iPSCs WT parental cell line | 1 | 4 | Organoids |  |  | |  | | 4 |
|  |  |  | i3N iPSCs MOCK Clone C3 | 1 | 2 | Organoids |  |  | |  | | 2 |
|  |  |  | i3N iPSCs SORL1 KO Clone D107 | 2 | 5 | Organoids |  |  | |  | | 5 |
|  |  |  | i3N iPSCs SORL1 p.Y1816C KI Clone F37 | 2 | 4 | Organoids |  |  | |  | | 4 |
|  | | | | | | | **D20** | | **D30** | | **D40** | |
| 3E-F | | TEM | i3N iPSCs WT parental cell line | 3 | 3 | iNeurons; coverslips |  | | 3 | |  | |
|  |  |  | i3N iPSCs SORL1 KO Clone D107 | 3 | 3 | iNeurons; coverslips |  | | 3 | |  | |
|  |  |  | i3N iPSCs SORL1 p.Y1816C KI Clone F37 | 3 | 3 | iNeurons; coverslips |  | | 3 | |  | |
| 4A-B | | PLA (IF) | i3N iPSCs WT parental cell line | 3 | 3 | iNeurons; coverslips |  | | 3 | |  | |
|  |  |  | i3N iPSCs SORL1 KO Clone D107 | 3 | 3 | iNeurons; coverslips |  | | 3 | |  | |
|  |  |  | i3N iPSCs SORL1 p.Y1816C KI Clone F37 | 3 | 3 | iNeurons; coverslips |  | | 3 | |  | |
| 4C | | ELISA | i3N iPSCs WT parental cell line | 6 | 8 | iNeurons; cell media |  | | 8 | |  | |
|  |  |  | i3N iPSCs MOCK Clone C3 | 4 | 4 | iNeurons; cell media |  | | 4 | |  | |
|  |  |  | i3N iPSCs MOCK Clone C11 | 4 | 4 | iNeurons; cell media |  | | 4 | |  | |
|  |  |  | i3N iPSCs SORL1 KO Clone D107 | 6 | 9 | iNeurons; cell media |  | | 9 | |  | |
|  |  |  | i3N iPSCs SORL1 KO Clone K4 | 3 | 3 | iNeurons; cell media |  | | 3 | |  | |
|  |  |  | i3N iPSCs SORL1 KO Clone K7 | 4 | 4 | iNeurons; cell media |  | | 4 | |  | |
|  |  |  | i3N iPSCs SORL1 p.Y1816C KI Clone F37 | 4 | 5 | iNeurons; cell media |  | | 5 | |  | |
|  | | | | | | | **D9** | **D15** | | **D30** | | **D60** |
| 4D-E; S4A | IHC | | i3N iPSCs WT parental cell line | 3 | 3 | Organoids; thick slices |  |  | |  | | 3 |
|  |  |  | i3N iPSCs SORL1 KO Clone D107 | 3 | 3 | Organoids; thick slices |  |  | |  | | 3 |
|  |  |  | i3N iPSCs SORL1 p.Y1816C KI Clone F37 | 2 | 2 | Organoids; thick slices |  |  | |  | | 2 |
| 4F | ELISA | | i3N iPSCs WT parental cell line | 4 | 9 | Organoids; media |  |  | |  | | 9 |
|  |  |  | i3N iPSCs MOCK Clone C3 | 3 | 8 | Organoids; media |  |  | |  | | 8 |
|  |  |  | i3N iPSCs MOCK Clone C11 | 2 | 3 | Organoids; media |  |  | |  | | 3 |
|  |  |  | i3N iPSCs SORL1 KO Clone D107 | 4 | 8 | Organoids; media |  |  | |  | | 8 |
|  |  |  | i3N iPSCs SORL1 KO Clone K4 | 3 | 8 | Organoids; media |  |  | |  | | 8 |
|  |  |  | i3N iPSCs SORL1 KO Clone K7 | 3 | 8 | Organoids; media |  |  | |  | | 8 |
|  |  |  | i3N iPSCs SORL1 p.Y1816C KI Clone F37 | 4 | 8 | Organoids; media |  |  | |  | | 8 |
|  | | | | | | | **D20** | | **D30** | | **D40** | |
| S4F | WB | | i3N iPSCs WT parental cell line | 3 | 1 | iNeurons; cell lysate |  | | 3 | |  | |
|  |  |  | i3N iPSCs MOCK Clone C3 | 3 | 1 | iNeurons; cell lysate |  | | 3 | |  | |
|  |  |  | i3N iPSCs MOCK Clone C11 | 3 | 1 | iNeurons; cell lysate |  | | 3 | |  | |
|  |  |  | i3N iPSCs SORL1 KO Clone D107 | 3 | 1 | iNeurons; cell lysate |  | | 3 | |  | |
|  |  |  | i3N iPSCs SORL1 KO Clone K4 | 3 | 1 | iNeurons; cell lysate |  | | 3 | |  | |
|  |  |  | i3N iPSCs SORL1 KO Clone K7 | 3 | 1 | iNeurons; cell lysate |  | | 3 | |  | |
|  |  |  | i3N iPSCs SORL1 p.Y1816C KI Clone F37 | 3 | 1 | iNeurons; cell lysate |  | | 3 | |  | |
|  | | | | | | | **D9** | **D15** | | **D30** | | **D60** |
| S4G | WB | | i3N iPSCs WT parental cell line | 2 | 1 | Organoids; cell lysate |  |  | |  | | 2 |
|  |  |  | i3N iPSCs MOCK Clone C3 | 2 | 1 | Organoids; cell lysate |  |  | |  | | 2 |
|  |  |  | i3N iPSCs MOCK Clone C11 | 2 | 1 | Organoids; cell lysate |  |  | |  | | 2 |
|  |  |  | i3N iPSCs SORL1 KO Clone D107 | 2 | 1 | Organoids; cell lysate |  |  | |  | | 2 |
|  |  |  | i3N iPSCs SORL1 KO Clone K4 | 2 | 1 | Organoids; cell lysate |  |  | |  | | 2 |
|  |  |  | i3N iPSCs SORL1 KO Clone K7 | 2 | 1 | Organoids; cell lysate |  |  | |  | | 2 |
|  |  |  | i3N iPSCs SORL1 p.Y1816C KI Clone F37 | 2 | 1 | Organoids; cell lysate |  |  | |  | | 3 |
| 5B-E | | SEM | i3N iPSCs WT parental cell line | 3 | 3 | iNeurons; coverslips |  | | 3 | |  | |
|  |  |  | i3N iPSCs SORL1 KO Clone D107 | 3 | 3 | iNeurons; coverslips |  | | 3 | |  | |
|  |  |  | i3N iPSCs SORL1 p.Y1816C KI Clone F37 | 3 | 3 | iNeurons; coverslips |  | | 3 | |  | |
| 5F-J; S5A-B; 6A-E; S6A-B | | Life Imaging (IF) | i3N iPSCs WT parental cell line | 3 | 6 | iNeurons; Ibidi 8 well |  | | 6 | |  | |
|  |  |  | i3N iPSCs SORL1 KO Clone D107 | 3 | 8 | iNeurons; Ibidi 8 well |  | | 8 | |  | |
|  |  |  | i3N iPSCs SORL1 p.Y1816C KI Clone F37 | 3 | 7 | iNeurons; Ibidi 8 well |  | | 7 | |  | |
|  | | | | | | | **D20** | | **D30** | | **D40** | |
| S5D | WB | | i3N iPSCs WT parental cell line | 1 | 1 | iNeurons; cell lysate |  | | 1 | |  | |
|  |  |  | i3N iPSCs SORL1 KO Clone D107 | 1 | 1 | iNeurons; cell lysate |  | | 1 | |  | |
|  |  |  | i3N iPSCs SORL1 p.Y1816C KI Clone F37 | 1 | 1 | iNeurons; cell lysate |  | | 1 | |  | |
| S5D | WB | | i3N iPSCs WT parental cell line | 1 | 1 | iNeurons; endosomes |  | | 1 | |  | |
|  |  |  | i3N iPSCs SORL1 KO Clone D107 | 1 | 1 | iNeurons; endosomes |  | | 1 | |  | |
|  |  |  | i3N iPSCs SORL1 p.Y1816C KI Clone F37 | 1 | 1 | iNeurons; endosomes |  | | 1 | |  | |
|  | | | | | | | **D9** | **D15** | | **D30** | | **D60** |
| S5E | WB | | i3N iPSCs WT parental cell line | 2 | 1 | Organoids; cell lysate |  |  | |  | | 2 |
|  |  |  | i3N iPSCs SORL1 KO Clone D107 | 2 | 1 | Organoids; cell lysate |  |  | |  | | 2 |
|  |  |  | i3N iPSCs SORL1 p.Y1816C KI Clone F37 | 2 | 1 | Organoids; cell lysate |  |  | |  | | 2 |
|  |  |  | i3N iPSCs WT parental cell line | 2 | 1 | Organoids; endosome |  |  | |  | | 2 |
|  |  |  | i3N iPSCs SORL1 KO Clone D107 | 2 | 1 | Organoids; endosome |  |  | |  | | 2 |
|  |  |  | i3N iPSCs SORL1 p.Y1816C KI Clone F37 | 2 | 1 | Organoids; endosome |  |  | |  | | 2 |
|  | | | | | | | **D20** | | **D30** | | **D40** | |
| S6C-E | | ELISA; WB; Life Imaging (IF) w BACEi | i3N iPSCs WT parental cell line | 2 | 3 | iNeurons; cell lysate; media; Ibidi 8 well |  | | 3 | |  | |
|  |  |  | i3N iPSCs SORL1 KO Clone D107 | 2 | 2 | iNeurons; cell lysate; media; Ibidi 8 well |  | | 2 | |  | |
|  | | | | | | | **D20** | | | | | |
| 7A-D | | MEA | i3N iPSCs WT parental cell line | 3 | 24 | iNeurons; MEA well | 24 | | | | | |
|  |  |  | i3N iPSCs MOCK Clone C3 | 3 | 24 | iNeurons; MEA well | 24 | | | | | |
|  |  |  | i3N iPSCs SORL1 KO Clone D107 | 3 | 24 | iNeurons; MEA well | 24 | | | | | |
|  |  |  | i3N iPSCs SORL1 KO Clone K4 | 3 | 24 | iNeurons; MEA well | 24 | | | | | |
|  |  |  | i3N iPSCs SORL1 p.Y1816C KI Clone F37 | 3 | 24 | iNeurons; MEA well | 24 | | | | | |
|  | | | | | | | **D20** | | **D30** | | **D40** | |
| S7 | WB | | i3N iPSCs WT parental cell line | 5 | 1 | iNeurons; cell lysate |  | | 5 | |  | |
|  |  |  | i3N iPSCs MOCK Clone C3 | 2 | 1 | iNeurons; cell lysate |  | | 2 | |  | |
|  |  |  | i3N iPSCs MOCK Clone C11 | 2 | 1 | iNeurons; cell lysate |  | | 2 | |  | |
|  |  |  | i3N iPSCs SORL1 KO Clone D107 | 5 | 1 | iNeurons; cell lysate |  | | 5 | |  | |
|  |  |  | i3N iPSCs SORL1 KO Clone K4 | 2 | 1 | iNeurons; cell lysate |  | | 2 | |  | |
|  |  |  | i3N iPSCs SORL1 KO Clone K7 | 2 | 1 | iNeurons; cell lysate |  | | 2 | |  | |
|  |  |  | i3N iPSCs SORL1 p.Y1816C KI Clone F37 | 5 | 1 | iNeurons; cell lysate |  | | 5 | |  | |

# **Table 3: Media, primers and antibodies used in this study.**

## **Inducible Neurons Induction Medium (IM)**

| **ITEM** | **COMPANY** | **IDENTIFIER** | **STORAGE CONCENTRATION** | **FINAL CONCENTRATION** |
| --- | --- | --- | --- | --- |
| DMEM/F12 | Thermo Fisher Scientific | Cat# 21331046 | Basal medium | Basal medium |
| HEPES | Thermo Fisher Scientific | Cat# 15630106 | 1M | 15mM |
| N-2 Supplement | Thermo Fisher Scientific | Cat# 17502048 | 100X | 1X |
| GlutaMAX™ | Thermo Fisher Scientific | Cat# 35050061 | 100X | 1X |
| MEM Non-Essential AA | Thermo Fisher Scientific | Cat# 11140035 | 100X | 1X |
| ZellShield® | Minerva Biolabs | Cat# 13-0050 | 100X | 0.5X |

## **Inducible Neurons Cortical Neuron Medium (CNM)**

| **ITEM** | **COMPANY** | **IDENTIFIER** | **STORAGE CONCENTRATION** | **FINAL CONCENTRATION** |
| --- | --- | --- | --- | --- |
| BrainPhys™ Neuronal Medium | STEMCELL Technologies | Cat# 5790 | Basal medium | Basal medium |
| B-27™ Supplement | Thermo Fisher Scientific | Cat# 17504044 | 50X | 1X |
| BDNF | PeproTech | Cat# 450-02 | 10 μg/mL | 10 ng/mL |
| NT-3 | PeproTech | Cat# 450-03 | 10 μg/mL | 10 ng/mL |
| Laminin Mouse Protein | Thermo Fisher Scientific | Cat# 23017015 | 1 mg/mL | 1 µg/mL |
| ZellShield® | Minerva Biolabs | Cat# 13-0050 | 100X | 0.5X |

## **Cerebral Organoids Neural Induction Medium (NIM)**

| **ITEM** | **COMPANY** | **IDENTIFIER** | **STORAGE CONCENTRATION** | **FINAL CONCENTRATION** |
| --- | --- | --- | --- | --- |
| DMEM/F12 | Thermo Fisher Scientific | Cat# 21331046 | Basal medium | Basal medium |
| N-2 Supplement | Thermo Fisher Scientific | Cat# 17502048 | 100X | 1X |
| GlutaMAX™ | Thermo Fisher Scientific | Cat# 35050061 | 100X | 1X |
| MEM Non-Essential AA | Thermo Fisher Scientific | Cat# 11140035 | 100X | 1X |
| Heparin sodium salt | Sigma | Cat# H3149 | 1 mg/mL (in H_2_O) | 1 µg/mL |
| 2-Mercaptoethanol | Sigma | Cat# M3148 | 10mM (in PBS) | 0.05mM |
| ZellShield® | Minerva Biolabs | Cat# 13-0050 | 100X | 1X |

## **Cerebral Organoid Differentiation Medium without vitamin A (CODM−A)**

| **ITEM** | **COMPANY** | **IDENTIFIER** | **STORAGE CONCENTRATION** | **FINAL CONCENTRATION** |
| --- | --- | --- | --- | --- |
| DMEM/F12 | Thermo Fisher Scientific | Cat# 21331046 | Basal medium | 1/2 of Basal medium |
| Neurobasal™ | Thermo Fisher Scientific | Cat# 21103049 | Basal medium | 1/2 of Basal medium |
| N-2 Supplement | Thermo Fisher Scientific | Cat# 17502048 | 100X | 0.5X |
| Insulin | Sigma | Cat# I9278 | 10 mg/mL | 2.5 µg/mL |
| GlutaMAX™ | Thermo Fisher Scientific | Cat# 35050061 | 100X | 1X |
| MEM Non-Essential AA | Thermo Fisher Scientific | Cat# 11140035 | 100X | 0.5X |
| B-27™ Supplement minus vitamin A | Thermo Fisher Scientific | Cat# 12587010 | 50X | 0.5X |
| 2-Mercaptoethanol | Sigma | Cat# M3148 | 10mM (in PBS) | 0.05mM |
| ZellShield® | Minerva Biolabs | Cat# 13-0050 | 100X | 1X |

## **Cerebral Organoid Differentiation Medium with vitamin A (CODM+A)**

| **ITEM** | **COMPANY** | **IDENTIFIER** | **STORAGE CONCENTRATION** | **FINAL CONCENTRATION** |
| --- | --- | --- | --- | --- |
| DMEM/F12 | Thermo Fisher Scientific | Cat# 21331046 | Basal medium | 1/2 of Basal medium |
| Neurobasal™ | Thermo Fisher Scientific | Cat# 21103049 | Basal medium | 1/2 of Basal medium |
| N-2 Supplement | Thermo Fisher Scientific | Cat# 17502048 | 100X | 0.5X |
| Insulin | Sigma | Cat# I9278 | 10 mg/mL | 2.5 µg/mL |
| GlutaMAX™ | Thermo Fisher Scientific | Cat# 35050061 | 100X | 1X |
| MEM Non-Essential AA | Thermo Fisher Scientific | Cat# 11140035 | 100X | 0.5X |
| B-27™ Supplement | Thermo Fisher Scientific | Cat# 17504044 | 50X | 0.5X |
| 2-Mercaptoethanol | Sigma | Cat# M3148 | 10mM (in PBS) | 0.05mM |
| ZellShield® | Minerva Biolabs | Cat# 13-0050 | 100X | 1X |

## **Chemicals**

| **ITEM** | **COMPANY** | **IDENTIFIER** | **STORAGE CONCENTRATION** | **FINAL CONCENTRATION** |
| --- | --- | --- | --- | --- |
| DMSO | Sigma | Cat# D2650 | 100% | 0.1% |
| LY2886721 BACE1 Inhibitor | Med Chem Express | Cat# HY-13240 | 25µM | 25nM |

## **CRISPR sequences**

| **CONSTRUCT** | **SEQUENCE 5’-3’** |
| --- | --- |
| SORL1-KO gRNA | ATTGAACGACATGAACCCTC |
| SORL1-KI p.Y1816C gRNA | GGCCCAGGCAGAAATCTCAT |
| SORL1-KI p.Y1816C ssODN | ACTTCCTCCTGGAATCTCCTTTTCCTGTTTTCACAGTTGGCAATCTGACAGCTCATACGTCATGTGAGATTTCCGCCTGGGCCAAGACTGACTTGGGGGATAGCCCTCTGGCATTTGAGCATGTTATG |
| SORL1- KO seq primer FW | TTCCTAAGCAGGGCAGGGTT |
| SORL1- KO seq primer RV | ATCTGTCCCAAGCACATCCT |
| SORL1- KI p.Y1816C seq primer FW | TACCCAGTGCTTGTGCAGT |
| SORL1- KI p.Y1816C seq primer RV | GGTTGATGGCTTTGGCCTTG |

## **PCR primers**

| **TARGET** | **FW SEQUENCE 5’-3’** | **RV SEQUENCE 5’-3’** |
| --- | --- | --- |
| *APOE2* | CGGACATGGAGGACGTGT | CTGGTACACTGCCAGGCA |
| *APOE3* | CGGACATGGAGGACGTGT | CTGGTACACTGCCAGGCG |
| *APOE4* | CTGGTACACTGCCAGGCG | CGGACATGGAGGACGTGC |
| *GDF15p* | ACACATCAAGGTTGCCCTTC | GGGCCTCAGTATCCTCTTCC |
| SORL1-KO OT1 | TTCGTGATCAGAGCACAGGG | TGCCAGATCCAGGGCTTTTA |
| SORL1-KI OT1 | AGAACAGCAAACAAACTAGGCAG | TCCTTTTCAGATGCATCACCCT |
| SORL1-KI OT2 | TTTGGCACAGACTCAAGCGA | GGGAGGGGCTTTCTCATTCC |
| SORL1-KI OT3 | AGCAAGGCTGCTAGTGTGAA | GCTGGTGCTCCACACAATTT |
| SORL1-KI OT4 | CTCCCCCAACAAAGATGGCA | CCCTGAGCTCAAACATGGGT |
| SORL1-KI OT5 | ACAGTCCTTTCCCTCTTAGCAG | AGGCATTTTGGAAGCGGATT |
| SORL1 Exon 41 skipping (E40-42) | TTCTGGGCATTTGACACCCA | AGCTCTTCGGGTTGCGATAG |

## **qPCR primers**

| **TARGET** | **NCBI GENE ID** | **FW SEQUENCE 5’-3’** | **RV SEQUENCE 5’-3’** |
| --- | --- | --- | --- |
| *GAPDH* | 2597 | AGCCACATCGCTCAGACAC | GCCCAATACGACCAAATCC |
| *OCT4* | 5460 | GAAACCCACACTGCAGATCA | CGGTTACAGAACCACACTCG |
| *NANOG* | 79923 | TTTGTGGGCCTGAAGAAAACT | AGGGCTGTCCTGAATAAGCAG |
| *SOX2* | 6657 | TACAGCATGTCCTACTCGCAG | GAGGAAGAGGTAACCACAGGG |
| *SOX1* | 6656 | TCCCCCGCGTGAACTG | CAAGGCATTTTGCGTTCACA |
| *PAX6* | 5080 | CTGAGGAATCAGAGAAGACAGGC | ATGGAGCCAGATGTGAAGGAGG |
| *TUBB3* | 10381 | TCAGCGTCTACTACAACGAGGC | GCCTGAAGAGATGTCCAAAGGC |
| *DCX* | 1641 | TATGCGCCGAAGCAAGTCTCCA | CATCCAAGGACAGAGGCAGGTA |
| *NEUN* | 146713 | TACGCAGCCTACAGATACGCTC | TGGTTCCAATGCTGTAGGTCGC |
| *MAP2* | 4133 | TTGGTGCCGAGTGAGAAGA | GTCTGGCAGTGGTTGGTTAA |
| *SYN1* | 6853 | AGTTCTTCGGAATGGGGTGAA | CAAACTGCGGTAGTCTCCGTT |
| *SYP* | 6855 | CTCGGCTTTGTGAAGGTGCT | CTGAGGTCACTCTCGGTCTTG |
| *PSD95* | 1742 | TCGGTGACGACCCATCCAT | GCACGTCCACTTCATTTACAAAC |
| *GRIA1* | 2890 | TGCTTTGTCGCAACTCACAGA | GGCATAGACTCCTTTGGAGAAC |
| *GRIN1* | 2902 | CTACCGCATACCCGTGCTG | GCATCATCTCAAACCACACGC |
| *GRIN2A* | 2903 | TCATGCAGGATTATGACTGGCA | TGTGGTCTTGACGAAGCTGAT |
| *GRIN2B* | 2904 | CAGTAGGGCTGACTGGCTATG | CAGGCACTGTGTCTGTATCCC |
| *TRKB* | 4915 | ACCCGAAACAAACTGACGAGT | AGCATGTAAATGGATTGCCCA |
| *TRKC* | 4916 | CTTTGCCCAGCCAAGTGTAGT | CGTGATGTTGATACTGGCGTT |
| *S100B* | 6285 | TGGCCCTCATCGACGTTTTC | ATGTTCAAAGAACTCGTGGCA |
| *GFAP* | 2670 | CCGACAGCAGGTCCATGT | GTTGCTGGACGCCATTG |
| *TMEM119* | 338773 | CTTCCTGGATGGGATAGTGGAC | GCACAGACGATGAACATCAGC |
| *AIF1* | 199 | TTGGTGAGAAACGGGTGATTTG | ATGGAGCATGTAGGAGAGACC |
| *PLP1* | 5354 | ACCTATGCCCTGACCGTTG | TGCTGGGGAAGGCAATAGACT |
| *OLIG2* | 10215 | ATGCACGACCTCAACATCGCCA | ACCAGTCGCTTCATCTCCTCCA |
| *SORL1* | 6653 | CCAACCTGAAGATGGGTCATA | ACAGCAGCAACATCCGTAGAT |
| *APP* | 351 | CAAGCAGTGCAAGACCCATC | AGAAGGGCATCACTTACAAACTC |

## **Antibodies**

| **ANTIBODY** | **COMPANY** | **IDENTIFIER** | **WB DILUTION** | **IF DILUTION** | **IHC DILUTION** |
| --- | --- | --- | --- | --- | --- |
| β-Actin | Cell Signaling Technology | Cat# 3700  RRID: AB_2242334 | 1:10,000 | - | - |
| LR11 (SORLA) | BD Biosciences | Cat# 611861  RRID: AB_399341 | 1:4,000 | - | - |
| OCT4 | Cell Signaling Technology | Cat# 2840  RRID: AB_2167691 | 1:1,000 | - | - |
| SOX2 | Cell Signaling Technology | Cat# 4900  RRID: AB_10560516 | 1:500 | - | - |
| SOX1 | Cell Signaling Technology | Cat# 4194  RRID: AB_1904140 | 1:1,000 | - | - |
| TUBB3 | Cell Signaling Technology | Cat# 5568  RRID: AB_10694505 | 1:1,000 | 1:200 | 1:200 |
| DCX | Santa Cruz Biotechnology | Cat# sc-271390  RRID: AB_10610966 | 1:300 | - | - |
| MAP2 | Cell Signaling Technology | Cat# 8707  RRID: AB_2722660 | 1:1,000 | - | - |
| NEUN | Cell Signaling Technology | Cat# 24307  RRID: AB_2651140 | 1:1,000 | - | - |
| SYN1 | Cell Signaling Technology | Cat# 5297  RRID: AB_2616578 | 1:1,000 | 1:300 | - |
| SYP | Thermo Fisher Scientific | Cat# PA1-1043  RRID: AB_2199026 | 1:300 | 1:200 | - |
| TRKB | Abcam | Cat# ab18987  RRID: AB_444716 | 1:1,000 | - | - |
| PSD95 | Cell Signaling Technology | Cat# 36233  RRID: AB_2721262 | 1:1,000 | - | - |
| HOMER1 | SYSY | Cat# 160003  RRID: AB_887730 | 1:1,000 | 1:200 | - |
| APP (Y188) | Abcam | Cat# ab32136  RRID: AB_2289606 | 1:1,000 | 1:500 | - |
| Anti-Mouse HRP | Cell Signaling Technology | Cat# 7076  RRID: AB_330924 | 1:3,000 | - | - |
| Anti-Rabbit HRP | Cell Signaling Technology | Cat# 7074  RRID: AB_2099233 | 1:3,000 | - | - |
| EEA1 | Thermo Fisher Scientific | Cat# 14-9114-82  RRID: AB_2572929 | - | 1:500 | 1:250 |
| β-Amyloid | Cell Signaling Technology | Cat# 8243  RRID: AB_2797642 | - | 1:200 | 1:200 |
| MAP2 | Milipore | Cat# AB5543  RRID: AB_571049 | - | 1:500 | - |
| NeuN | Cell Signaling Technology | Cat# 94403  RRID: AB_2904530 | - | - | 1:200 |
| Alexa Fluor 594 | Invitrogen | Cat# A21203  RRID: AB_141633 | - | 1:500 | 1:400 |
| Alexa Fluor 488 | Life Technologies | Cat# A11008  RRID: AB_143165 | - | 1:500 | 1:200 |
| Alexa Fluor 647 | Jackson Im.Research | Cat# 703-606-155  RRID: AB_2340380 | - | 1:500 | 1:400 |
