# Supplementary material for: SORL1 variant links endosomal amyloid precursor protein mis‐sorting to axonal transport defects and neuronal dysfunction
Source: Alzheimers Dement. 2026 Aug 22;22(8):e71782. doi: 10.1002/alz.71782 (PMC13498896; doi:10.1002/alz.71782)
Supplement: Supplementary file 3 [file ALZ-22-e71782-s003.docx]

**Supplementary materials and methods**

**Cell culture and gene editing**

A human iPSC line with a stably integrated, doxycycline-inducible NGN2 transgene (i3N iPSCs, RRID: CVCL_C7XJ) was used in this study. The i3N cell line was generously provided by Michael Ward.^34^ Cells were maintained as a monolayer on Matrigel® hESC-qualified matrix (Corning) in mTeSR™1 medium (STEMCELL Technologies) supplemented with 1X Zellshield™ (Minerva Biolabs) and passaged using TrypLE™ Express reagent (Thermo Fisher Scientific). IPSCs were maintained at 37 °C in a 5% CO_2_ incubator.

Three clones of *SORL1* Knock-Out (KO) and one clone of heterozygous p.Y1816C Knock-In (KI) iPSCs used in this study were generated using a CRISPR/Cas9 ribonucleoprotein (RNP) complex containing Cas9-GFP (Sigma). For *SORL1*-KO, exon 6 of the *SORL1* gene in i3N iPSCs was targeted using a previously published guide RNA (gRNA)^19^. The p.Y1816C KI was introduced by targeting exon 41 with a custom-designed gRNA and a single-stranded oligodeoxynucleotide (ssODN) as a template for homology-directed repair, as published previously ^35^. CRISPR/Cas9 RNP complexes were delivered using the Neon™ Transfection System (Thermo Fisher Scientific), followed by GFP-based single-cell sorting into 96-well plates to generate clonal lines. *SORL1*-KO clones were initially screened by Western blotting for the absence of SORLA protein by LR11 antibody (BD Transduction Laboratories) and confirmed by Sanger sequencing (Seqme). The *SORL1*-KI clone was initially screened by PCR-RFLP. PCR product with WT sequence was cleaved by BtsCI (New England Biolabs), while PCR product with the introduced p.Y1816C mutation was cleaved by AciI (New England Biolabs) and confirmed by Sanger sequencing (Seqme). To control for the potential effects of the genome editing process itself, two MOCK control clones were generated by subjecting the parental WT iPSC line to the complete CRISPR workflow, including transfection, sorting, and clonal expansion, without introducing any targeted modification. All CRISPR-edited cell lines were confirmed to have an unchanged karyotype by G-banding (Cytogenetic Laboratory Brno, Ltd.). All CRISPR-edited cell lines were screened by Sanger sequencing for predicted off-target sites with up to three mismatches (CHOP-CHOP). *SORL1*-KI clone was screened for skipping of exon 41 by RT-PCR/PCR. Unless otherwise stated, all experiments were performed using three clones of WT iPSCs (one parental cell line and two MOCK clones), three clones of KO iPSCs, and one clone of KI iPSCs. Clone 1 of each genotype is represented by circles, clone 2 by squares, and clone 3 by triangles in the graphs.

A comprehensive list of all used cell lines and clones is provided in **Table S1**; a list of all experimental samples, including iPSC clones used for individual analyses and the number of biological replicates, is provided in **Table S2;** and sequences of gRNAs, ssODNs, and PCR primers are provided in **Table S3**.

**Differentiation of 2D neurons and 3D cerebral organoids**

The parental iPSC line used in this study carries a doxycycline-inducible NGN2 cassette, which was used only for the generation of 2D induced neurons. Cerebral organoids were generated without doxycycline exposure using a patterning-based differentiation protocol; therefore, NGN2 was not intentionally induced during organoid differentiation.

2D Neuronal differentiation of i3N iPSCs was performed using a previously published protocol ^34^. Briefly, undifferentiated iPSCs (Day 0) were plated as a single-cell monolayer on Matrigel®-coated dishes at a density of 0.7 × 10⁵ cells/cm² in Induction Medium (IM) supplemented with 2 μg/mL doxycycline (Sigma) and 10 μM Y-27632 ROCK inhibitor (Selleckchem). From Day 1 to Day 2, IM containing 2 μg/mL doxycycline was changed daily. On Day 3, cells were replated onto Poly-L-Ornithine-coated dishes (Sigma) using Accutase (Thermo Fisher Scientific) at a density of 0.8 × 10⁵ cells/cm² in Cortical Neuron Medium (CNM). Half of the CNM was replaced twice per week until sample collection. Samples were harvested on Days 20, 30, and 40. For the collection of conditioned media for ELISA and Western blot analyses, CNM was replaced with Essential 6 medium (Thermo Fisher Scientific), and cells were cultured for an additional 3 days prior to sampling. Media composition is provided in **Table S3.**

3D cerebral organoids were generated using a modified protocol from ^36^ as previously described by Vanova *et al.* ^37^ without a doxycycline induction step. Briefly, on Day 0, iPSC lines were dissociated using Accutase (Thermo Fisher Scientific) and seeded into non-adherent, V-bottom 96-well plates at a density of 2,000 cells per well in 150 µl of mTeSR™1 medium (STEMCELL Technologies) supplemented with 50 μM Y-27632 (Selleckchem) to initiate embryoid body (EB) formation. The next day, the medium was changed to fresh mTeSR without Y-27632 (100 µl per well). Once EBs reached a diameter of ≥400 μm, fresh Neural Induction Medium (NIM) was added daily for 6 days (typically from Day 3 to Day 8, 100 µl per well). After this induction phase, organoids were embedded in 7 μL of cold Geltrex™ (Thermo Fisher Scientific). Following polymerization, the Geltrex droplets containing organoids were gently detached and cultured without agitation on Petri dishes in Cerebral Organoid Differentiation Medium without vitamin A (CODM−A) for 7 days. Organoids were then transferred to Cerebral Organoid Differentiation Medium with vitamin A (CODM+A) and placed on an orbital shaker between Days 24 and 28. Half of the medium was replaced three times per week. Samples were collected on Days 9, 15, 30, and 60. For whole cell lysate and RNA isolation, 5 to 10 organoids were pooled per sample. For ELISA, individual organoids were cultured in 12-well plates with 0.8-1.5 mL of Essential 6 medium (Thermo Fisher Scientific) for 72 hours prior to the collection of conditioned media and organoids. Media compositions are provided in **Table S3.**

**BACE1 Inhibition**

Inducible neurons were seeded at a density of 60,000 cells per well in 8-well multichannel slide (Ibidi) for live imaging and 300,000 cells per well in 12-well plate (TPP) for Western blotting and ELISA. After 14 days of maturation in CNM medium, cells were treated with either 25 nM of the β-secretase inhibitor LY2886721 (BACEi; MCE) or DMSO (vehicle control) for 7 days, with daily half-media changes. Cells for live imaging of axonal transport were transfected on day 17 with the APP-GFP lentiviral particles (Flash Therapeutics) described in the section “Live imaging and axonal transport analyses”. On day 21, after 8 days of drug treatment, samples were collected for Western blotting and ELISA, and live imaging of APP-GFP was performed.

**DNA Isolation, PCR and Sanger Sequencing**

Genomic DNA was isolated using the DNeasy Blood & Tissue Kit (Qiagen) according to the manufacturer’s instructions. PCR amplification was performed with Taq DNA polymerase (Top-Bio). PCR amplification was performed under the following conditions: 94 °C/3 min; 27 cycles of (94 °C/30 s, 65 °C/30 s, 72 °C/1 min); 72 °C/10 min; 4 °C. PCR for Apolipoprotein E (*APOE*) polymorphisms was performed using an allele-specific PCR conditions as previously described ^38^. Visualization of PCR products was performed by electrophoresis on a 2% agarose gel containing GelRed® (Biotium) and visualized using the ChemiDoc™ Touch Imaging System (Bio-Rad). *GDF15p* was used as a loading control. PCR products for sequencing were purified using QIAquick PCR Purification Kit (Qiagen) and sequenced by Sanger sequencing (SEQme, Ltd.). Primer sequences are listed in **Table S3.**

**RNA isolation, cDNA synthesis, and qPCR**

Total RNA was extracted using RNA Blue reagent (Top-Bio) following the manufacturer’s protocol. RNA concentration and purity were assessed using a NanoDrop 1000 spectrophotometer (Thermo Fisher Scientific). Reverse transcription was performed with 0.5–1 μg of total RNA using the Transcriptor First Strand cDNA Synthesis Kit (Roche), according to the manufacturer’s instructions. Quantitative PCR (qPCR) was carried out using the LightCycler® 480 SYBR Green I Master Kit (Roche) in 10 μL reactions on a LightCycler® 480 II system (Roche). The thermal cycling protocol included an initial denaturation at 95 °C for 5 min, followed by 45 amplification cycles (95 °C for 10 s, 60 °C for 10 s, and 72 °C for 10 s). Threshold cycle (Ct) values were determined using the Second Derivative Maximum Method in the LightCycler® 480 software. Gene expression was analyzed using the ΔCt method (Ct_target – Ct_housekeeping) and reported as 2^–ΔCt. Primer sequences are listed in **Table S3**.

**Western blotting and isolation of endosomes**

Cells were washed with ice-cold PBS and lysed on ice for 1 hour in lysis buffer (20 mM Tris-HCl, pH 8.1; 10 mM EDTA; 1% Triton X-100; 1% NP-40) supplemented with cOmplete™ Mini Protease Inhibitor (Roche). Samples were sonicated by SonoPuls HD 2200 (power 20%; pulse 1 s; time 10 s; Bandelin). Protein concentrations were determined using the DC™ Protein Assay Kit (Bio-Rad). Equal amounts of protein were mixed with 4× Laemmli buffer, heated at 95 °C for 10 minutes, and separated by SDS-PAGE using custom-cast acrylamide gels. Proteins were transferred to PVDF membranes (Merck). Membranes were blocked for 1 hour in blocking buffer (5% skim milk in TBS containing 0.1% Tween-20), followed by overnight incubation at 4°C with primary antibodies diluted in blocking buffer. The following day, membranes were incubated with HRP-conjugated secondary antibodies for 1 hour at room temperature. Signals were detected using Clarity™ and Clarity Max™ ECL substrates (Bio-Rad) and visualized with the ChemiDoc™ Touch Imaging System (Bio-Rad). Band intensities were quantified using Image Lab software (Bio-Rad). A complete list of antibodies is provided in **Table S3.**

To analyze endosome-associated cargo in 2D neurons and 3D cerebral organoids, endosomal fractions were isolated using the Trident Endosome Isolation Kit (GeneTex) according to the manufacturer’s protocol. For each sample, approximately 10 million cells were used for endosome isolation. In parallel, whole-cell lysates were prepared from the same differentiation batch to allow comparison between total protein abundance and endosome-enriched protein abundance. Isolated endosomal fractions were lysed and processed using the same western blotting workflow as described for whole-cell lysates.

**ELISA**

Conditioned cell culture media samples were centrifuged at 10,000 × g for 5 minutes, and the resulting supernatant was collected, supplemented with cOmplete™ Mini Protease Inhibitor (Roche), and stored at –80 °C until ELISA analysis. Media volumes (V) were measured at the time of collection to account for the variable rate of evaporation between wells, and the values were used for ELISA normalization. Whole-cell lysates were prepared by incubating cells on ice for 1 hour in lysis buffer (20mM Tris-HCl, pH 8.1; 10mM EDTA; 1% Triton X-100; 1% NP-40) supplemented with cOmplete™ Mini Protease Inhibitor (Roche). Total protein concentration in the lysates was determined using the DC Protein Assay Kit (Bio-Rad), multiplied by lysate volume to obtain total protein mass (m), and used to normalize ELISA results. Levels of Aβ peptides (Aβ40 and Aβ42) in conditioned media were quantified using the Amyloid Beta 40 Human ELISA Kit and the Amyloid Beta 42 Human ELISA Kit, Ultrasensitive (both Thermo Fisher Scientific), according to the manufacturer’s protocols. SORLA levels in conditioned media were measured using the Human SORL1 ELISA Kit (Abcam). All samples were analyzed in technical duplicates. To enable comparison across samples, values obtained from ELISA (c) were normalized to total protein content and media volume using the formula (c*V)/m and are expressed as picograms (pg) of analyte per microgram (µg) of total protein.

**Immunocytochemistry and image analysis**

For 2D immunofluorescence, iPSC-derived neurons were seeded at a density of 75,000 cells per well on glass coverslips (Thermo Fisher Scientific). Cells were fixed in 4% paraformaldehyde (PFA) for 20 minutes, permeabilized for 15 minutes with 0.1% Triton X-100, and blocked for 1 hour in 1% BSA and 0.03% Tween-20 in PBS. Primary antibodies diluted in blocking buffer were applied overnight at 4 °C, followed by incubation with secondary antibodies for 1 hour at room temperature. Nuclei were counterstained with DAPI (Sigma), and coverslips were mounted using ProLong™ Gold Antifade mounting medium (Thermo Fisher Scientific).

For thick-section immunostaining of Aβ clusters in 3D organoids, harvested samples were fixed with 4% PFA for 1 hour at room temperature. Fixed cerebral organoids were embedded in 4% agarose and sectioned into 200 μm sections using a vibratome (Leica). Sections were incubated for 4 hours at room temperature in blocking buffer containing 0.5% Triton X-100, 5% BSA, and 0.01% sodium azide in PBS. Primary antibodies diluted in blocking buffer were applied for 4 days at 4 °C, followed by incubation with secondary antibodies for 24 hours at 4 °C. Nuclei were counterstained with DAPI, and sections were cleared using a glycerol/fructose solution to enhance imaging depth and quality.

For cryosection immunostaining of neuronal markers and early endosomes in 3D organoids, harvested samples were fixed with 4% PFA for 1 hour at room temperature. Fixed organoids were cryoprotected in 30% sucrose, embedded in OCT, and sectioned at 20 μm using a Leica CM1850 cryostat. Sections were washed in PBS to remove residual OCT, permeabilized for 10 minutes with 0.2% Triton X-100, and blocked for 1 hour in a solution containing 1% BSA, 0,03% Tween-20 and 0,01% sodium azide. Primary antibodies were applied overnight at 4 °C, followed by incubation with secondary antibodies for 1 hour at room temperature. Nuclei were counterstained with DAPI, and sections were mounted using ProLong Gold Antifade mounting medium prior to imaging.

Images of 2D neurons and organoid cryosections were acquired using a Zeiss Axio Observer.Z1 microscope equipped with an LSM800 confocal unit using a Plan-Apochromat 63×/1.40 oil immersion objective. Thick organoid sections were imaged using a Plan-Apochromat 20×/0.8 air objective. For colocalization analysis, images were acquired using an IXplore SpinSR SoRa confocal microscope equipped with a 60x oil-immersion objective and a 3.2x SoRa magnification disk.

For endosome size analysis, confocal z-stacks were imported into Fiji/ImageJ and converted into maximum intensity projections. In 2D neurons, a MAP2-derived neuronal mask was generated and applied to restrict the analysis to neuronal structures. The EEA1 channel was background-corrected using a rolling ball algorithm, thresholded, converted to a binary mask, and processed using the Watershed algorithm. Automated particle analysis was performed on objects with an area between 0.02 and 1µm². A total of 28-33 images per genotype from three biological replicates were analyzed. For organoid cryosections, the same analysis pipeline was used, except that no MAP2 masking was applied. For endosome analysis in organoid cryosections, 5–7 images per genotype from two independent biological replicates were analyzed.

Colocalization analysis was performed in Fiji/ImageJ on confocal Z-stacks. Neuronal regions were identified using a MAP2-derived maskand fluorescence intensities of the red and green channels were quantified following background subtraction. Colocalization was assessed using tresholded Mander’s overlap coefficient. In total, 24 images per genotype were analyzed from one biological replicate.

Aβ volume measurements in vibratome-sectioned organoids were performed using IMARIS software version 10 (Oxford Instruments). The “Surfaces” tool was used to assess Aβ size, with volume selected as the measurement parameter. Thresholds were optimized using control samples and subsequently applied uniformly to all datasets. For data analysis, a total of 108 images per genotype were examined in three biological replicates.

**Proximity Ligation Assay – PLA**

iPSC-derived neurons were seeded at a density of 75,000 cells per well on glass coverslips (Thermo Fisher Scientific). Cells were fixed, permeabilized, and blocked as described above for immunocytochemistry and processed using the Duolink® In Situ Red Starter Kit Mouse/Rabbit (Sigma) according to the manufacturer’s instructions. Primary antibodies were applied overnight at 4 °C, followed by incubation with PLA probes, and subsequent ligation and amplification steps. Cells were mounted using Duolink® In Situ Mounting Medium with DAPI and imagined within two days to ensure signal preservation. Microscopy was performed using Zeiss Axio Observer.Z1 microscope equipped with an LSM800 confocal unit and a Plan-Apochromat 63×/1.40 oil immersion objective. Image analysis was performed using Fiji/ImageJ software using the automated particle analysis. For data analysis, a total of 39 images per genotype were analyzed from three independent biological replicates.

**Transmission electron microscopy – TEM**

iPSC-derived neurons were seeded at a density of 300,000 cells per well on a 6-well plate. After 20 days of maturation in CNM medium, samples were fixed in 3% glutaraldehyde (Sigma) in 0.1 M cacodylate buffer (pH 7.3; Sigma) with 2% saccharose and 1% tannin for 24 hours at 4 °C. After washing by 0.1M cacodylate buffer samples were post-fixed in 1% OsO4 (Sigma) in the same buffer for 1 hour at room temperature, following by dehydration ethanol series (50%, 70%, 96% and 100% ethanol) and embedded in the LR White resin (Agar Scientific), polymerization for 3 days at 65 °C. The resin blocks were processed using the standard protocol for electron microscopy, with cutting performed on a LEICA EM UC6 ultramicrotome (Leica). The ultrathin sections were examined using Morgagni 268D transmission electron microscope (Thermo Fisher Scientific), working at 90 kV and equipped with Veleta CCD camera (Olympus). For data analysis, a total of 60 images per genotype were examined. This included three biological replicates per genotype, with 20 images analyzed per replicate. The diameter of the endosomes was measured using the iTEM acquisition software (Olympus).

**Scanning electron microscopy - SEM**

iPSC-derived neurons were seeded at a density of 45,000 cells per well on glass coverslips (Thermo Fisher Scientific) placed in 24-well plates. After 20 days of maturation in CNM medium, wells were washed with PBS and fixed in 3% glutaraldehyde (Sigma) in 0.1 M cacodylate buffer (pH 7.3; Sigma) with 2% saccharose for 24 hours at 4 °C. Samples were washed with 0.1 M cacodylate buffer containing 2% saccharose and dehydrated in a series of increasing ethanol concentrations (30%, 50%, 70%, 96%, and 100% ethanol). They were then dried in a Critical Point Dryer (Bal-Tec CPD 030, LEICA). After that, samples were fixed on stubs and sputtered with gold using Q 150RS plus coater (Quorum Technologies). The samples were examined using TESCAN VEGA TS 5136 XM scanning electron microscope (TESCAN) at high vacuum mode, secondary electron detector mode, accelerating voltage of 15 kV at 3,000x magnification.

For data analysis, approximately five projections per image were analyzed (>10 images per biological replicate). For each projection, the width and length of swellings, as well as the width and length of the shaft, were measured using ImageJ software. Minor shaft enlargements (<200% of shaft width), branching points, and enlargements at crossings were excluded. The number of swellings was then calculated per 100 µm of shaft length.

**Live imaging and axonal transport analyses**

iPSC-derived neurons were seeded at a density of 60,000 cells per well in 8-well ibidi multichannel slide (Ibidi). Neurons were transduced at Day 20 with APP-GFP lentiviral particles (Flash Therapeutics) and with Bacmam 2.0 RFP-Rab5a (Thermo Fisher Scientific), both previously used in Feole *et al.* ^24^. Particles were resuspended in CNM according to the validated multiplicity of infection (MOI; APP-GFP = MOI 5; RFP-Rab5a = MOI 10) and transduction units per milliliter (TU/mL) provided by the manufacturer. They were then incubated with cells for 24 hours, after which fresh CNM media was added. Transduction efficiency was monitored every 48 hours until the start of live-cell imaging experiments after 10 days of transduction (Day 30 of differentiation). Prior to acquisition, the CNM medium was replaced with artificial Cerebrospinal Fluid solution (aCSF) at pH 7.4 to avoid any interference from the phenol red contained in the original culture medium. Time-lapse recordings of axonal transport were performed in all genotypes for a total duration of 60 s, with varying frame rates depending on the cargo. Movement of APP-GFP vesicles was recorded at 2 frames per second (fps), while that of Rab5a vesicles was recorded at 1 fps. Recordings were obtained using a Zeiss LSM 780 microscope equipped with an LSM7 Live module, an incubation system to maintain cultures at 37 °C and 5% CO_2_, and acquisition was carried out with 63×/1.4 NA Plan Apochromat oil-immersion objective (Zeiss) to achieve maximum resolution.

For the following axonal transport analyses, the recorded movies were first processed in ImageJ and subsequently analyzed in Imaris v.10 (Oxford Instruments). Particle trajectories were identified using either the semi-automated “Spots” (APP-GFP) or “Surfaces” (RFP-Rab5a) tracking tool integrated with an Autoregressive Motion algorithm, for segmentation and thresholding of the APP and Rab5a particles. Input parameters included empirically determined XY diameters, maximum displacement distance, and maximum allowed gap size, tailored to the spatial and temporal resolution of each dataset. Key parameters used to assess axonal transport dynamics included track displacement (t*Dx*), defined as the spatial difference between the starting and ending *x*-coordinates of a particle (t*Dx*(t_1_, tₙ) = P*x*(tₙ) – P*x*(t₁) – where P indicates the position), and track duration (t*d*), indicating the total time a particle remained in motion. Tracks shorter than 10 seconds were excluded from further analysis. Based on average velocity, particles were classified as stationary or moving, and moving tracks were further categorized as anterograde or retrograde according to directionality. For APP-GFP, particles covering total distances > 250 nm were considered moving particles. If moving at average velocities of either ≥ 0.1 µm/s or -≤0.1 µm/s, they were considered fast anterograde and retrograde, respectively. All Rab5a-RFP particles, regardless of the speed, with a total track displacement ≥ 250 nm were considered in movement; otherwise, they were considered stationary. Track length was determined from the vectorial displacement (*x* and *y* dimensions) computed by the analysis software. Additionally, since movement of Rab5+ endosomes was analyzed using the “Surfaces” tool, the algorithm also allowed for the quantification of endosomal size over time, using the same time-lapse recordings acquired for axonal transport analysis.

**Electrophysiology**

iPSC-derived neurons were seeded at a density of 20,000 cells/well in the Poly-L-Ornithine/Laminin-coated 96-well MEA plates (Axion BioSystems). Each cell clone was seeded in 8 separate wells per individual run. Neurons were maintained in CNM for a period of 8 weeks. Spontaneous neuronal activity was recorded every other day for 5 minutes at 37 °C using the Axion Maestro system with the Middleman interface (Axion Biosystems). Raw electrophysiological data were processed using the Neural Metrics Tool in AxiS 2.4 software (Axion Biosystems) and exported for further analysis. Activity detection parameters were defined as follows: (a) an electrode was considered active if it recorded >1 spike per minute; (b) spike detection threshold was set at 6.5 standard deviations above the baseline noise; and (c) bursts were defined as a minimum of 5 spikes with an inter-spike interval no greater than 100 ms. To assess network maturation and activity, the following five parameters were analyzed: (1) weighted mean firing rate per well (average firing rate across active electrodes), (2) number of active electrodes per well, (3) percentage of spikes occurring within bursts, (4) number of bursts per well, and (5) synchrony index, representing the temporal correlation of firing across electrodes in a well.

**Data analysis**

Data are presented as mean ± standard error of the mean (SEM). Normality of data distribution was assessed prior to statistical testing. Parametric tests (t-test or one-way ANOVA) were applied to normally distributed data, while non-parametric tests (Mann–Whitney U test or Kruskal–Wallis test) were used for non-normally distributed data when comparing two or three groups, respectively. Statistical analyses were performed using GraphPad Prism version 9.0.0 for Windows (GraphPad Software; www.graphpad.com). A p-value ≤ 0.05 was considered statistically significant.
