# Supplementary material for: SORL1 variant links endosomal amyloid precursor protein mis‐sorting to axonal transport defects and neuronal dysfunction
Source: Alzheimers Dement. 2026 Aug 22;22(8):e71782. doi: 10.1002/alz.71782 (PMC13498896; doi:10.1002/alz.71782)
Supplement: Supplementary file 4 [file ALZ-22-e71782-s004.docx]

**Supplementary Figures**

**Figure S1**

**Figure S2**

**Figure S3**

**Figure S4**

**Figure S5**

**Figure S6**

**Figure S7**

**Supplementary Figure legends**

**Figure S1: Genetic validation and characterization of *SORL1* gene-edited iPSC lines. (A)** Karyotype analysis showing a normal chromosomal profile of all gene-edited lines. **(B)** APOE genotyping shows a homozygous APOE3/3 genotype in i3N iPSC line. **(C)** PCR/ELFO shows KO-target site minor rearrangements in KO clones. **(D)** Sanger sequencing showing introduction of the heterozygous *SORL1* p.Y1816C point mutation in the KI line. **(E)** Sanger sequencing showing off-target sites of all gene-edited lines. **(F)** PCR/ELFO shows no exon skipping in the KI line of exon 41 (containing p.Y1816C point mutation).

**Figure S2. Supporting data for neuronal marker expression, SORLA levels, and sSORLA detection in neurons and organoids. (A, B)** Additional validation of progenitor and neuronal marker expression at the mRNA and protein levels in 2D neuron cultures across WT, KO, and KI lines. **(C, D)** Gene and protein expression analysis of pluripotency, progenitor, neuronal, synaptic, and glial markers in cerebral organoids. **(E)** Western blot analysis of culture medium from day 60 cerebral organoids of sSORLA in KO and KI-derived organoids compared to WT; **(F)** Densitometric quantification of sSORLA levels from (E). Each dot represents a biological replicate (n ≥ 2); Error bars represent mean ± SEM. Statistical significance was determined using an unpaired statistical test. *p < 0.05, **p < 0.01, WT vs KO = * WT vs KI = #

**Figure S3. Analyses of endosomal morphology and spatial distribution across genotypes.** **(A)** Classification of endolysosomal structures identified in TEM images, including endocytosis of clathrin-coated pits and vesicles, electron-lucent (EL) endosomes, electron-dense (ED) endosomes, and vesicles undergoing exocytosis; **(B)** Representative TEM fields showing clustering of enlarged endosomes within the neuronal soma in WT, KO, and KI neurons; TEM scale bar: 500 nm (A) 1000 nm (B).

**Figure S4. Additional analyses of Aβ cluster size and APP expression in neurons and organoids. (A)** Median volume of Aβ particles in cerebral organoids across WT, KI, and KO genotypes; **(B)** APP mRNA expression in neurons and organoids across WT, KI, and KO lines, as measured by qRT-PCR. **(C)** Representative Western blot and densitometric quantification of full-lenght APP (APP-FL) in inducible neurons across WT, KO, and KI lines during maturation. **(D)** Representative Western blot and densitometric quantification of full-length APP (APP-FL) in cerebral organoids across WT, KO, and KI lines during maturation. **(E)** Representative Western blot and densitometric quantification of β-CTFs/APP-FL ratio in inducible neurons across WT, KO, and KI lines at D30. **(F)** Representative Western blot and densitometric quantification of β-CTFs/APP-FL ratio in cerebral organoids across WT, KO, and KI lines at D60. Each dot represents one biological replicate (n ≥ 3). Data represent mean ± SEM; significance determined by an unpaired statistical test; ***p < 0.001.

**Figure S5. Supplementary quantification of Rab5a-positive endosome dynamics in WT, KI, and KO axons. (A)** Proportion of Rab5a-RFP particles moving anterogradely, retrogradely, or remaining stationary in WT, KI, and KO neurons, quantified per axon; **(B)** Velocity of moving Rab5a-RFP particles tracked over time (0–60 s) for anterograde and retrograde directions across genotypes. Each dot represents one axon. Total of 20 axons were measured in three independent biological replicates. Each dot represents one particle **(C)** Lenght of track of individual Rab5a-RFP particle in WT, KI, and KO neurons. Each dot represents one particle. Total of 20 axons were measured in three independent biological replicates. **(D)** Representative Western blot of DCTN1 in the whole cell lysate and endosomal fraction of neurons at D30 and densitometric quantification of DCTN1 normalized on GAPDH (whole cell lysates) and amidoblack staining (endosomal fractions). **(E)** Representative Western blot of DCTN1 in the whole cell lysate and endosomal fraction of cerebral organoids at D60 and densitometric quantification of DCTN1 in cerebral organoids normalized on GAPDH (whole cell lysates) and EEA1 (endosomal fractions). Data represent mean ± SEM; significance determined an unpaired statistical test; *p < 0.05, ***p < 0.001, ****p < 0.0001.

**Figure S6. Additional transport metrics of APP-GFP.** **(A)** Proportion of APP-GFP particles moving anterogradely, retrogradely, or remaining stationary in WT, KI, and KO neurons, quantified per axon. Each dot represents one axon. Total of 20 axons were measured in three independent biological replicates. **(B)** Lenght of track of individual APP-GFP particle in WT, KI, and KO neurons. Each dot represents one particle. Total of 20 axons were measured in three independent biological replicates. **(C)** Representative immunofluorescence images and quantification of co-localization of EEA1-positive early endosomes with APP-FL and Amyloid-β in neurites (MAP2 positive) of 2D neurons at day 30. **(D)** ELISA measurement of secreted Aβ40 and Aβ42 levels in 2D neuronal culture media with DMSO and BACE1 inhibitor. **(E)** Representative Western blot and densitometric quantification of full-lenght APP (APP-FL) in inducible neurons treated with DMSO and BACE1 inhibitor. **(F)** Proportion of APP-GFP particles moving anterogradely, retrogradely, or remaining stationary in WT and KO neurons treated with DMSO and BACE1 inhibitor, quantified per axon. Each dot represents one axon. Total of 13 axons were measured in two independent biological replicates. Data represent mean ± SEM; significance determined an unpaired statistical test; *p < 0.05, **p < 0.01. Immunofluorescence scale bar: 4 µm

**Figure S7. Synaptic receptors quantification in neurons.** Representative Western blot and densitometric quantification of total level of presynaptic receptors (SYN1; SYP) and postsynaptic receptors (PSD95; HOMER1) in 2D neurons at day 30. Each dot represents one biological replicate (n ≥ 5). Data represent mean ± SEM; significance determined an unpaired statistical test; *p < 0.05, **p < 0.01.
